# Valorization of Poly(lactic acid) to Lactate Esters Using Task-Specific Ionic Liquids

**DOI:** 10.1021/acssuschemeng.5c08785

**Published:** 2025-11-21

**Authors:** Salvatore Marullo, Martina Silaco, Francesca D’Anna

**Affiliations:** Dipartimento STEBICEF, 18998 Università degli Studi di Palermo, Viale delle Scienze, Ed. 17, 90128 Palermo, Italy

**Keywords:** ionic liquids, plastic waste recycling, PLA, alcoholysis, lactate esters

## Abstract

This work proposes a methodology based on the use of acidic task-specific ionic liquids (TSILs) to obtain lactate esters from poly­(lactic acid) (PLA). TSILs differing for both the cation and the anion were synthesized, featuring imidazolium, ammonium, piperidinium, and morpholinium cations, bearing a solfobutyl chain. For anions, besides chloride, HSO_4_^–^ was also used. TSILs were prepared under microwave irradiation and applied considering the sustainability guidelines to minimize the environmental impact of the upcycling process. After optimizing experimental conditions for reaction time and temperature, catalyst loading, nucleophile amount, catalyst reuse and scale-up of the process were performed, processing a 5-fold higher polymer amount. Considering the industrial applications of lactate esters, we tested the general applicability of our methodology using different alcohols as nucleophiles. Results evidence the promising performance of aliphatic TSILs that at 100 °C, under autogenous pressure, allowed the obtainment of different lactate esters from PLA, with quantitative conversion and yield from 47 to 88%. The process can be easily scaled up, and the best catalyst can be reused for at least three cycles without significant performance loss. Results collected were evaluated by the holistic approach to Green Chemistry and compared with IL-catalyzed processes reported in the literature.

## Graphical abstract

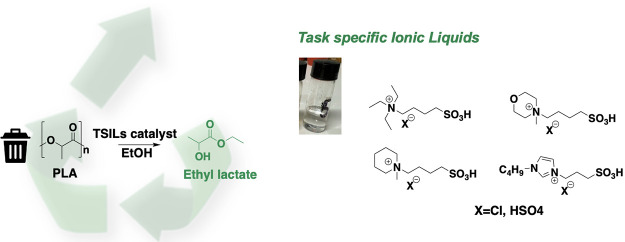

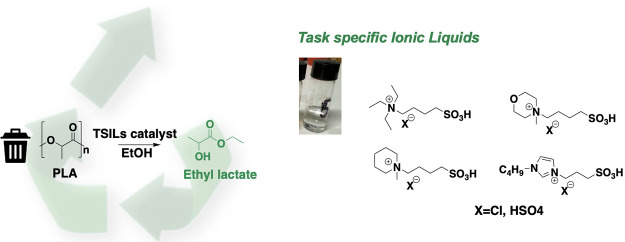

## Introduction

The advent of Green Chemistry has allowed the optimization of processes in the chemical industry, with the aim to counteract resource depletion and climate change issues. During the years, this approach has favored the transition from the linear economy to the circular economy model, rendering mandatory the circularity of materials to increase resource efficiency. In this context, plastics play a pivotal role. Indeed, they are ubiquitous in modern daily life, thanks to important features like cheapness, lightweight, and durability that allow their application in various sectors, such as food packaging, biomedical devices, and electronics.^1^ However, plastics currently represent an environmental issue that needs attention^2^ as their production annually achieves 300 million tons, consuming ca. 6% of oil produced globally that is estimated to rise to 20% by 2050.^3^ In addition, the plastic industry follows a linear model, and about 60% of production is discarded and accumulated in landfills or in the natural environment, where it degrades, forming micro- and nanoplastics. These particles are pervasive in the aquatic environment and pose significant risks to both ecosystem and human health. On this subject, different toxicological studies have demonstrated that they cause reactive oxygen species (ROS) generation, inflammation, and neurological dysfunction, alongside long-term risks such as immune disruption, cancer, and infertility.^4,5^ This is the reason why, in recent years, many researchers have turned their attention to the topic of plastic recycling.^6,7^

Chemical recycling of polymers represents a valid way to avoid the above issues as it takes advantage of chemical transformation (for example, hydrolysis and transesterification) to recover the pristine monomer (closed loop)^8,9^ or convert it directly into other value-added chemicals (open loop).^10,11^

On the other hand, a different strategy that can help to deal with the problem of plastic waste is the production of biobased polymers and degradable plastics. Among biobased polymers, poly­(lactic acid) (PLA) has been proposed as the best replacement for petroleum-based polyesters, and its production represents 32% of global biodegradable plastics. PLA is a renewable and biodegradable aliphatic polyester derived from the microbial fermentation of starch-rich feedstocks such as corn and sugar. Unfortunately, the degradation of PLA occurs very slowly in the real environment, both in seawater and in soil, producing CO_2_.^12,13^ This is the reason why, in accordance with the principles of circular economy, also in this case, valorization of PLA waste could represent a valuable way to avoid accumulation into the environment.

Among strategies aimed at the obtainment of value-added chemicals, transesterification to lactic esters is worth mentioning. This has received increasing attention since lactic esters represent a promising alternative to traditional petroleum-based solvents, thanks to their inherent biodegradability, low toxicity, and low vapor pressure.^14^ They possess excellent solvent properties, being able to dissolve oils, gums, dyes, synthetic polymers, and paints.^15^ Beside their use as green solvents, lactate esters have been also recently proposed as floatation frothers.^16^ Indeed, thanks to the presence of a polar head and an alkyl tail, they can act as cosurfactants. Propyl lactate and butyl lactate are also used in inks, fine chemicals, and paintings. Furthermore, in high purity, they are also used to produce chiral intermediates for pesticides and drugs.^17^ On the other hand, lactate esters can be reconverted into lactide, closing the loop in circular economy.^18,19^ All of the above considerations justify the surge of interest toward their obtainment also using waste as raw materials.

In the literature, a great deal of attention has been particularly devoted to methanolysis of PLA. This process frequently requires the use of metallic or corrosive catalysts, high temperature, and long reaction time^20,21^ and has been performed using catalysts of different nature, like commercial metal­[bis­(trimethylsilyl) amides],^22^ zirconium phosphate-supported WOx,^23^ multifunctional organofluoride catalysts,^24^ NaOH,^25^ superbases,^26,27^ and so on.

With the aim to overcome some of the above drawbacks, the use of ionic liquids (ILs) has also been taken in consideration. In this context, investigations so far reported have demonstrated the high efficiency of imidazolium-based ILs, bearing simple anions like acetate,^28^ or more complex species formed in the presence of metal chlorides or metal acetates.^29,30^

With the above information in mind and in the framework of our interest in studying plastic waste recycling,^13,31–33^ in this paper, we considered the acid-catalyzed alcoholysis of PLA using task-specific ionic liquids (TSILs) as catalysts. TSILs conjugate the advantages of ILs, such as low vapor pressure and flammability, with the advantages of supported catalysts. Indeed, they bear on the cation or anion structure the catalytic functionality that allows them to behave both as solvents and as catalysts.^34^ Generally, they can be easily reused, like supported catalysts, but with respect to the latter, they show higher kinetic mobility that boosts processes.

In this work, TSILs differing for the cationic head were considered, using both aromatic and aliphatic cations, to test catalysts of different eco- and cytotoxicities (Scheme 1).

**Scheme 1:**
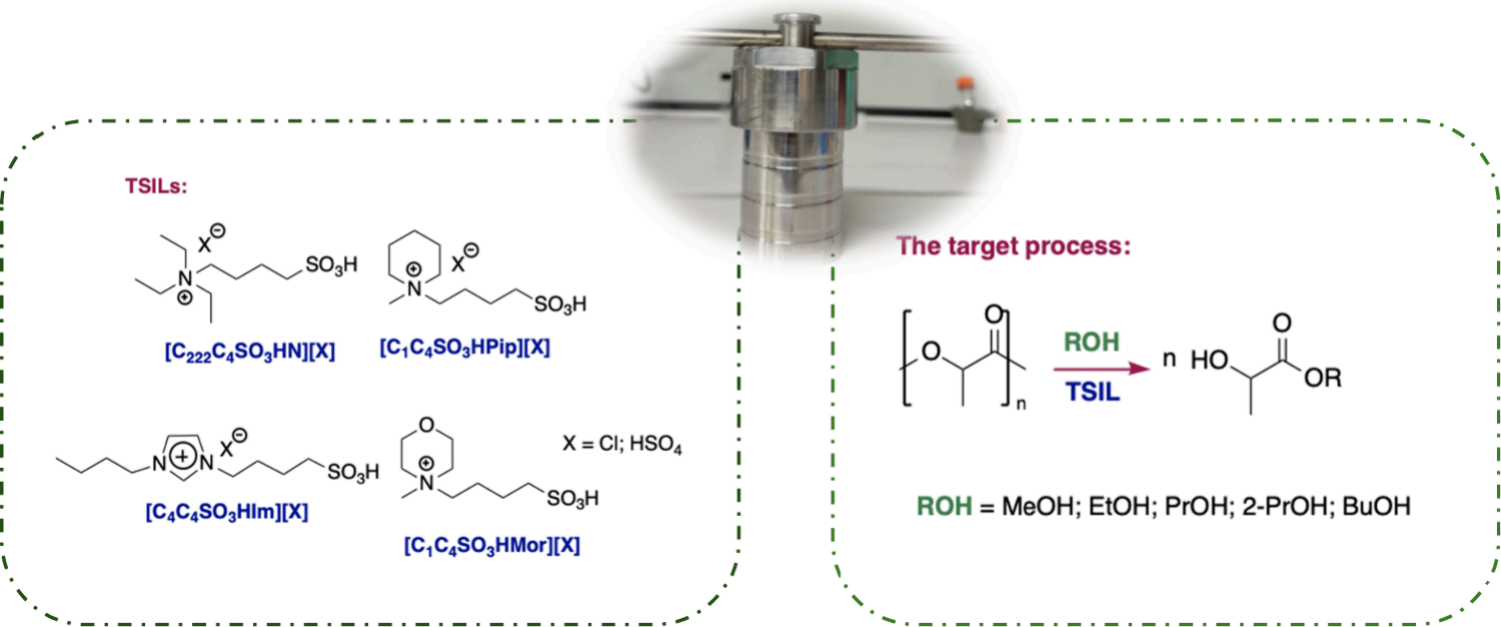
Schematic Representation of the Process and Catalysts Used

To address Green Chemistry guidelines, catalysts were synthesized under microwave (MW) irradiation to decrease the energy demand of the process.

Among aliphatic cations, ammonium, piperidinium, and morpholinium-based catalysts were used. All of these cations bear a solfobutyl chain, providing the catalysts with the needed acidic function. However, they were tested both as chloride and [HSO_4_^–^]-based ILs to modulate the acidic strength. The anchoring of acidic functionality on the cation or anion structure makes the catalyst easy to handle with respect to the corresponding mineral acids, also giving the possibility of reusing. In addition, using an inexpensive source like sulfuric acid to introduce the anion can contribute to lower the cost of these ILs.^35^

The process was first optimized using [C_1_C_4_SO_3_HPip]­[HSO_4_] as the catalyst and ethanol as the nucleophile, changing the reaction time and temperature, and analyzing the effect of the mass ratio of alcohol/PLA and catalyst loading. Then, under the best reaction conditions, the influence of the catalyst nature was evaluated, and for the best catalyst, the possible reuse and the effect of the different nature of the nucleophile were evaluated. Importantly, the process was also scaled up, processing a 5-fold higher amount of polymer without any loss in performance.

Data collected show that our catalysts allow the obtainment of lactate esters from PLA with high conversions and yields ranging from 52 up to 88%. The best performing catalyst can be reused for at least three cycles, and the proposed methodology can be successfully scaled up.

## Experimental Section

### Materials

*N-*Methyl morpholine, *N-*methylmorpholine, 1-methyl imidazole, triethylamine, 1,4-butane sultone, ethanol, methanol, 1-propanol, 2-propanol, and PLA were obtained from commercial sources and used without further purification.

Microwave-assisted synthesis was carried out with a CEM Discover oven. ^1^H and ^13^C NMR spectra were recorded using a Bruker 400 MHz spectrometer. FT-IR spectra were recorded, as KBr pellets or liquid films on an Agilent spectrophotometer.

### General Procedure for the Synthesis of Zwitterionic Compounds under Conventional Heating

Two grams of 1,4-butane sultone (15 mmol) was suspended in 10 mL of 2-propanol. To the resulting mixture was added dropwise a solution of the stoichiometric amount of the suitable amine dissolved in 5 mL of 2-propanol. The ensuing mixture was then refluxed with stirring for 24 h. Subsequently, the solvent was removed at reduced pressure, resulting in a light-orange solid. This solid was washed by sonicating it for 5 min with portions of ethyl acetate (3 × 8 mL), and removing the supernatant. The residual solvent was then removed at reduced pressure, obtaining a white solid, which is the zwitterion.

[C_4_C_4_SO_3_HIm]­[Cl] and [C_4_C_4_SO_3_HIm]­[HSO_4_] were prepared by following a literature procedure.^36^

### General Procedure for Microwave-Assisted Synthesis of Zwitterionic Compounds

500 mg of 1,4-butane sultone, the stoichiometric amount of the suitable amine, and 4 mL of 2-propanol were mixed in a closed vessel and irradiated with a microwave at 80 °C at a maximum power of 200 W for 2 h under stirring. Workup of the reaction was conducted as already described for the conventional synthesis.

### General Procedure for the Synthesis of TSILs

Two g of the suitable zwitterions were dissolved in 5 mL of MeOH/H_2_O 90 vol % mixture and then added to the stoichiometric amount of hydrochloric or sulfuric acid. The reaction mixture was stirred at room temperature overnight. Subsequently, evaporation of the solvent at reduced pressure yielded the TSILs as viscous pale-yellow oils.

### General Procedure for the Ethanolysis of PLA

In a typical experimental procedure, PLA pellets (0.5 g; PLA), ethanol (2 g), and catalyst (*n*_c_/*n*_RU_ = 0.10) were put in the PFTE chamber of a hydrothermal stainless-steel reactor, which was placed in the oven at set temperature for a suitable time. At the end of the reaction, the reactor was allowed to cool down to room temperature. Subsequently, the unreacted PLA was filtered off, dried, and weighed to determine the conversion. The filtered mixture was concentrated under reduced pressure to remove the alcohol, and the residual transparent liquid (containing the product, catalyst, and oligomers) was washed with hexane (5 mL). The hexane phase was transferred to a round-bottom flask and evaporated at reduced pressure, yielding ethyl lactate as a colorless liquid. The same procedure was followed for the depolymerization of PLA with other alcohols.

Conversions and yields were calculated according to the following equations:

$$\mathrm{Conversion}\;(\%)=\frac{\mathrm{PLA}\;(\mathrm{initial}\;\mathrm{mass})-\mathrm{PLA}\;(\mathrm{residual}\;\mathrm{mass})}{\mathrm{PLA}\;(\mathrm{initial}\;\mathrm{mass})}\times 100$$

$$\mathrm{Yield}\;(\%)=\frac{\mathrm{Obtained}\;\mathrm{mass}\;\mathrm{of}\;\mathrm{EL}}{\mathrm{Theoretical}\;\mathrm{mass}\;\mathrm{of}\;\mathrm{EL}}\times 100$$

For the catalyst recycling test, after the first reaction, the same workup procedure already described was applied. After the removal of hexane, the remaining residue was constituted by the catalyst. The latter was then charged with a fresh batch of PLA and ethanol, and the reaction was carried out as already described. This procedure was repeated until a 20% yield reduction was observed.

## Results and Discussion

### Synthesis of TSILs

TSILs used to catalyze the ethanolysis of PLA were prepared by a two-step procedure. In the first step, the amine was reacted with 1,4-butane sultone to obtain the zwitterion that, in the second step, was reacted with the equivalent amount of HCl or H_2_SO_4_ to obtain target TSILs in good yields (Scheme 2).

**Scheme 2:**
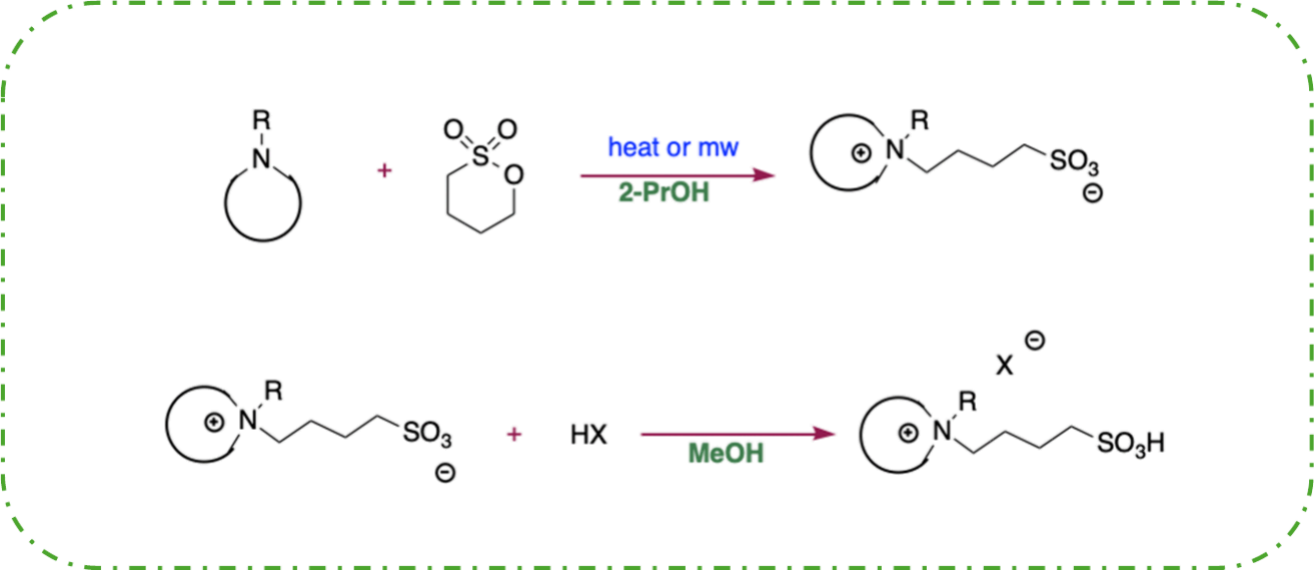
Schematic Representation of the TSIL Synthesis

It is noteworthy that, for both reactions, alcohols were used as solvents which, according to solvent selection guides, have a lower environmental impact and are also suitable to be used in larger-scale processes.^37^

To optimize TSIL synthesis, [C_1_C_4_SO_3_Mor] was used as a model compound, and the reaction was performed using a stoichiometric amount of 1,4-butane sultone. In this condition, in 2-propanol, a yield of 65% was obtained after both 24 and 48 h at 90 °C. With the aim to increase the yield, the amount of butane sultone was doubled, obtaining a yield in product equal to 77%. Under the same experimental conditions, [C_222_C_4_SO_3_N], [C_1_C_4_SO_3_Pip], and [C_4_C_4_SO_3_Im] were obtained with 65, 81, and 79% yields (Table 1).

**Table 1:** Yield Values for the Synthesis of the Zwitterions of TSILs in 2-PrOH Solution under Thermal Heating (24 h, 90 °C)

thermal heating	
zwitterion	yield (%)^a^
[C_1_C_4_SO_3_Mor]	77
[C_1_C_4_SO_3_Pip]	81
[(C_2_)_3_C_4_SO_3_N]	65
[C_1_C_4_SO_3_Im]	79

a Yields were reproducible within ±3%.

Considering the significant amount of energy used in such a process, the attempt to perform the reaction under MW irradiation was carried out. The temperature was set up at 80 °C and, operating at 200 W for 2 h, the yield in product gradually increased from 41 up to 85%, in parallel with the increase in the butane sultone amount from 1.2 up to 2 equiv (Table 2). Under the same experimental conditions, [C_222_C_4_SO_3_N], [C_1_C_4_SO_3_Pip], and [C_4_C_4_SO_3_Im] were collected with yields ranging from 85 up to 98% (Table 2).

**Table 2:** Yield Values for the Synthesis of the Zwitterions of TSILs in 2-PrOH Solution under MW Irradiation

MW irradiation		
zwitterion	reaction conditions	yield (%)^a^
[C_1_C_4_SO_3_Mor]	200 W, 2 h, 1 eq.	41
[C_1_C_4_SO_3_Mor]	200 W, 2 h, 2 eq.	85
[C_1_C_4_SO_3_Pip]	200 W, 2 h, 2 eq.	98
[(C_2_)_3_C_4_SO_3_N]	200 W, 2 h, 2 eq.	94
[C_1_C_4_SO_3_Im]	300 W, 2 h, 2 eq.	70

a Yields were reproducible within ±3%.

In all cases, the excess of 1,4-butanesultone was removed by washing the crude product with ethyl acetate, largely admitted by Green Chemistry on the grounds of the solvent selection guides.^37^ Then, keeping constant the amount of reagent and performing the reaction under MW irradiation allowed a decrease in the reaction temperature and a more significant drop in the reaction time.

### Optimization of the Experimental Conditions for the Ethanolysis of PLA

Considering the significantly high acidity of TSILs used as catalysts, in a first attempt, the ethanolysis of PLA was performed under atmospheric pressure but without success. Consequently, the process was always performed in a hydrothermal reactor at autogenous pressure. The reactor during the process was kept in an oven equipped with temperature control.

At the end of the reaction, the residual PLA was filtered under gravity, and after evaporation of the remaining alcohol at reduced pressure, the residue containing the catalyst and ethyl lactate was washed with the lowest amount of organic solvent to separate the product from the catalyst (Figure S5). To this aim, the possible use of solvents having low environmental impact and toxicity, like AcOEt and 2-Me-THF, generally recommended by solvent selection guides, was considered.^37^ Furthermore, also the use of diethyl ether and methyl isobutyl ketone was attempted. Unfortunately, in all cases, in the ^1^H NMR spectra of the isolated product, signals ascribable to the catalyst were observed, revealing product contamination. After different attempts, hexane was identified as the solvent able to recover EL and catalyst with high purity.

We are aware of both environmental and safety concerns for this solvent, and this is the reason why experiments aimed at minimizing the amount of solvent used were performed, using at the most 5 mL of hexane for 500 mg of PLA processed.

To set up the optimal experimental conditions, [C_1_C_4_SO_3_HPip]­[HSO_4_] was used as a model catalyst in the presence of EtOH. In a first attempt, the reaction was carried out for 2 h at 100 °C using a catalyst loading equal to 10% (*n*_c_/*n*_RU_ = moles of catalyst/number of repeating units in the polymer; where *n*_RU_ = mPLA/72) and a mass ratio of PLA/EtOH equal to 0.25. Under these experimental conditions, a quantitative conversion of PLA (*C* = 100%) was obtained with a yield in EL equal to 35%, indicating that the catalyst was able to induce the polymer breakdown into oligomers without achieving a full conversion of the latter into the corresponding monomer (Table S1).

To improve the above result, the amount of nucleophile was increased using a PLA/EtOH weight ratio equal to 0.17, with no significant changes in the yield value (40%; Table S1). However, notwithstanding the average yield, in both cases, a highly pure product was obtained, as accounted for by the absence of spurious signals in the ^1^H NMR spectrum (Figure S1).

Encouraged by the above results, the catalyst loading was increased up to 15%, and a significant increase in yield, equal to 51%, was observed after 2 h (Table S1). Consequently, according to the fifth Principle of Green Chemistry, which recommends limiting also the amount of auxiliary species, the effect of the reaction time was investigated, with no further increase in the amount of catalyst. On the other hand, reasoning in terms of scale-up and taking into consideration the acidic nature of the catalyst, employing a lower amount of catalyst should improve both safety and economical aspects, satisfying two of three requirements of sustainable development. Furthermore, from a chemical point of view, a higher catalyst loading could also be detrimental for the process as the increased acidity of the medium could also promote the product hydrolysis.

According to such considerations, the process was carried out increasing the reaction time up to 4 h, observing a gradual increase in yield from 51 up to 87% (Figure 1 and Table S2).

**Figure 1:**
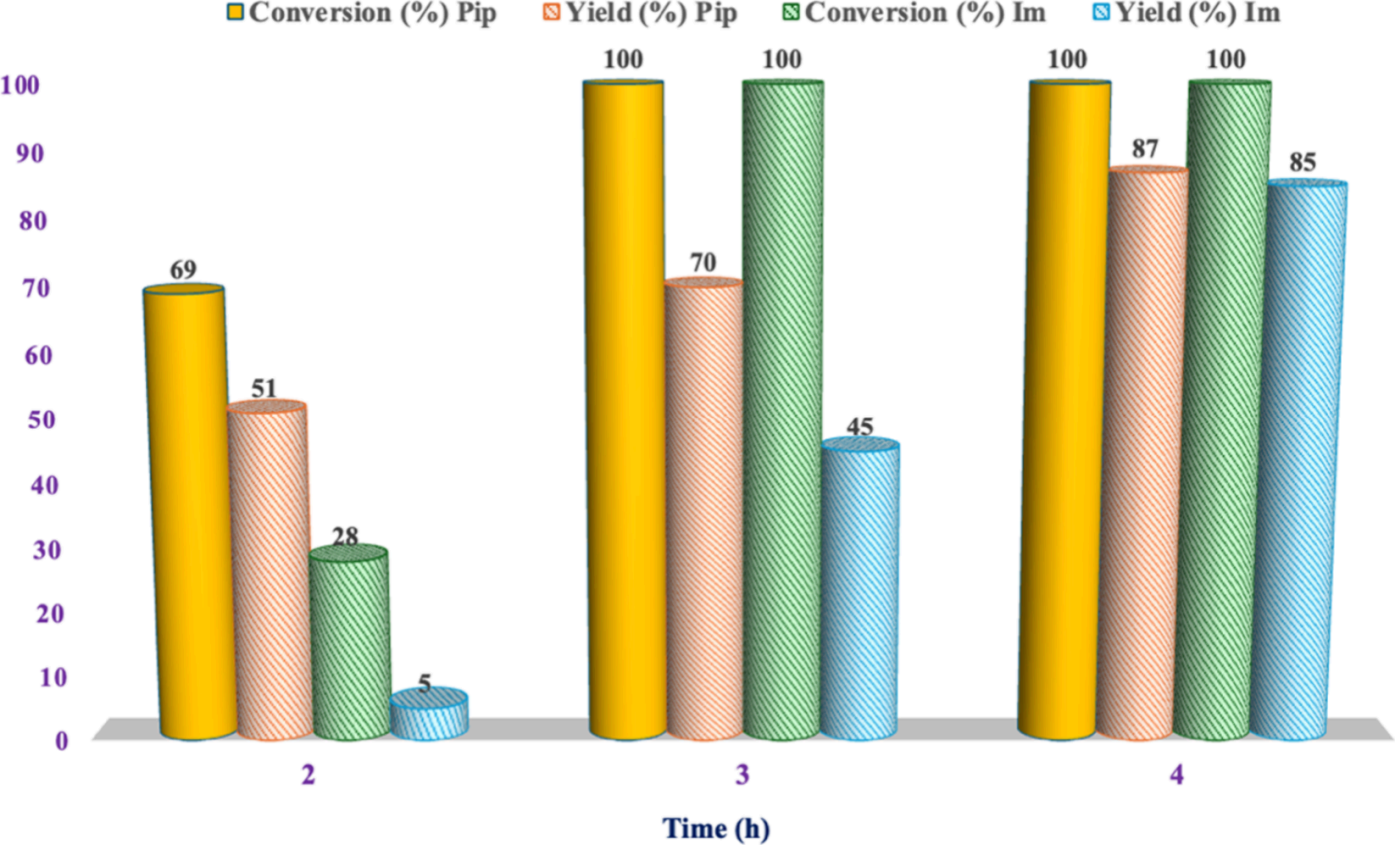
Conversion and yield values for the ethanolysis of PLA in the presence of [C_1_C_4_SO_3_HPip]­[HSO_4_] or [C_4_C_4_SO_3_HIm]­[HSO_4_] as the catalyst at 100 °C (*n*_c_/*n*_RU_ = 0.15; *m*_PLA_/*m*_EtOH_ = 0.25).

With the aim to have a first evaluation of the effect deriving from the nature of the cationic head of the catalyst, keeping constant all of the other experimental conditions, the same investigation was also carried out in the presence of [C_4_C_4_SO_3_HIm]­[HSO_4_] (Figure 1). Interestingly, it was observed that, in general, the aliphatic catalyst showed better performance than the corresponding aromatic one. Indeed, at lower reaction times (2 and 3 h), conversions obtained in the presence of [C_1_C_4_SO_3_HPip]­[HSO_4_] were comparable or higher with respect to the ones collected using [C_4_C_4_SO_3_HIm]­[HSO_4_]. Accordingly, the yields in EL were always higher in the presence of the first catalyst. After 4 h, two catalysts showed comparable performance, probably indicating a higher rate enhancement occurring in the presence of the aliphatic catalyst. Finally, to investigate if the reaction temperature could be decreased without compromising the yields, we carried out the reaction for 4 h at 90 °C. However, in this condition, the conversion dropped dramatically to 18% without obtaining any significant EL formation.

Since one of the goals of this work was the development of a methodology to perform alcoholysis of PLA under mild conditions, the following optimization was carried out at 100 °C without further raising the reaction temperature.

### Effect of Catalyst Nature

Having identified the optimal reaction time and temperature (100 °C, 4 h), the effect deriving from the different nature of the catalyst was analyzed. To this aim, different aliphatic TSILs catalysts were used, like ammonium- and morpholinium-based catalysts, besides the piperidinium one already described. We devoted our attention to aliphatic TSILs, to fulfill the third Green Chemistry Principle, since previously reported papers have largely demonstrated the lower cyto- and ecotoxicities of ILs bearing aliphatic cations compared with the relevant aromatic ones.^38^

In addition, the performance of chloride-based TSILs was also assessed to have information on the efficiency of less acidic catalysts. Results collected are reported in Figure 2 and Table S3.

**Figure 2:**
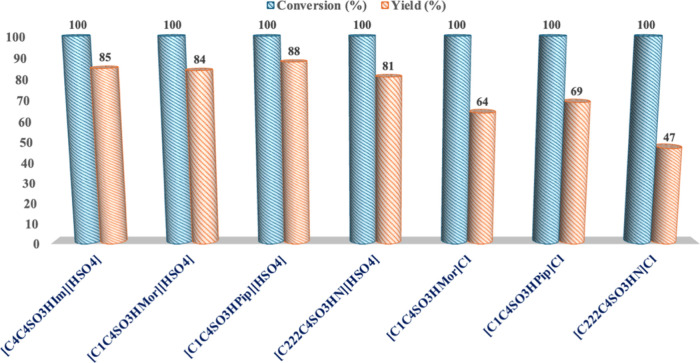
Conversion and yield values for the ethanolysis of PLA in the presence of different catalysts at 100 °C and for 4 h under autogenous pressure (*n*_c_/*n*_RU_ = 0.15; *m*_PLA_/*m*_EtOH_ = 0.25).

As far as the effect of the cation nature is concerned, using [HSO_4_^–^]-based catalysts, the data collected revealed the comparable performance of the catalysts used. Indeed, with the only exception of [(C_222_C_4_SO_3_HN]­[HSO_4_] for which a slightly lower yield value (81%) was found, in all cases, quantitative conversion with yields ranging from 84 ([C_1_C_4_SO_3_HMor]­[HSO_4_]) up to 88% ([C_1_C_4_SO_3_HPip]­[HSO_4_]) was observed. On the use of chloride-based TSILs, significantly lower yields were obtained. Indeed, in this case, yields ranged from 47 ([(C_222_C_4_SO_3_HN]­Cl) up to 69% ([C_1_C_4_SO_3_HPip]­Cl), evidencing the ability of these catalysts to favor the polymer breakdown in the oligomers without promoting the full depolymerization into the lactate ester. It is worth mentioning that in the case of chloride-based ILs, the reaction in the presence of [C_4_C_4_SO_3_HIm]Cl was also carried out for a longer time, 5 h. However, despite quantitative conversion, the presence of oligomers in the product was still observed.

Among the used catalysts, [C_1_C_4_SO_3_HMor]­[HSO_4_] was identified as the most suitable catalyst for the target process. Indeed, notwithstanding that it gave conversion and yield values comparable to the ones collected in the presence of all the other catalysts bearing the same anion, it possesses the cationic head with the lowest cyto- and ecotoxicities.^38^

### Effect of the Alcohol Nature

To obtain different lactate esters and assess the effect of alkyl chain elongation, we also investigated the trend of the target process as a function of the alcohol nature. Indeed, it is worth mentioning that, according to the holistic approach to Green Chemistry, the larger the applicability of a synthetic protocol, the higher the sustainability of a process.^39^ Besides EtOH, MeOH, PrOH, and BuOH were used. In the case of PrOH, the effect of the isomerism of the alkyl chain was evaluated, also using 2-PrOH. Data collected are displayed in Figure 3 and Table S4.

**Figure 3:**
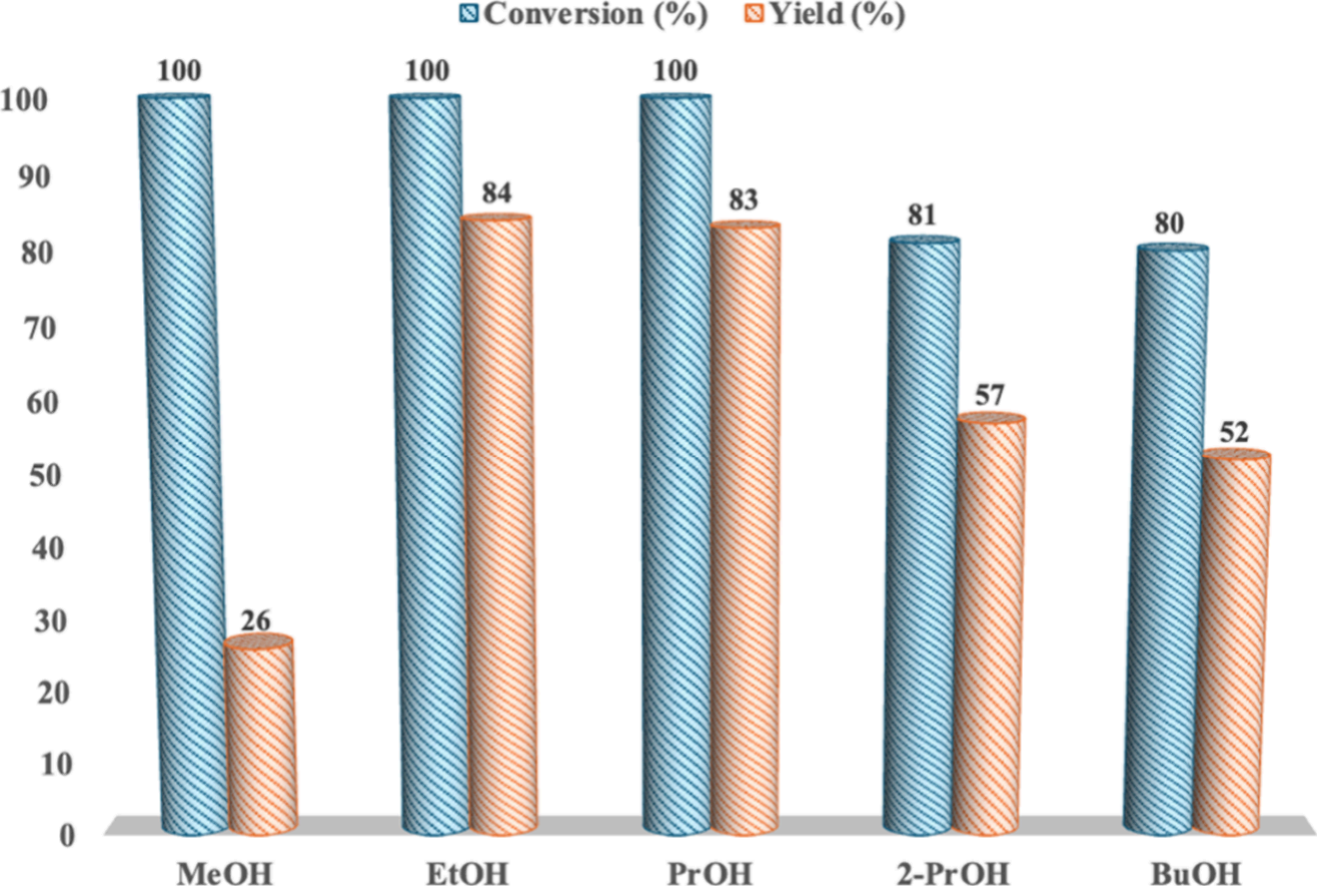
Conversion and yield values for the alcoholysis of PLA, performed in the presence of [C_1_C_4_SO_3_HMor]­[HSO_4_], at 100 °C and for 4 h under autogenous pressure, as a function of the different nature of the nucleophile (*n*_c_/*n*_RU_: 0.15; *m*_PLA_/*m*_RtOH_: 0.25).

Analysis of collected results evidences that the nature of the nucleophile significantly affects the outcome of the process. Indeed, quantitative conversions were obtained on going from MeOH up to PrOH. On the other hand, the use of a secondary alcohol (2-PrOH), the elongation of the alkyl chain, and the increase in solvent viscosity (BuOH) induced a significant decrease in the above parameter. With regard to yield values, after a first increase recorded in the presence of EtOH and PrOH, a regular decrease was observed as a result of both alkyl chain elongation (going from EtOH to BuOH) and branching (PrOH vs 2-PrOH). All these results allowed us to identify EtOH as the best nucleophile but at the same time indicate the general applicability of the methodology. In the case of MeOH, the low yield value could be a consequence of the higher solubility of the ester in the reaction mixture containing the TSIL with respect to the hexane phase.

### Catalyst Reuse and Scale-Up of the Process

With the best conditions at hand, the reuse of the catalyst was attempted. To this aim, catalysts that exhibited good performance, namely, [C_1_C_4_SO_3_HPip]­Cl, [C_1_C_4_SO_3_HPip]­[HSO_4_], and [C_1_C_4_SO_3_HMor]­[HSO_4_], were considered. Data collected are reported in Figure 4 and Table S5.

**Figure 4:**
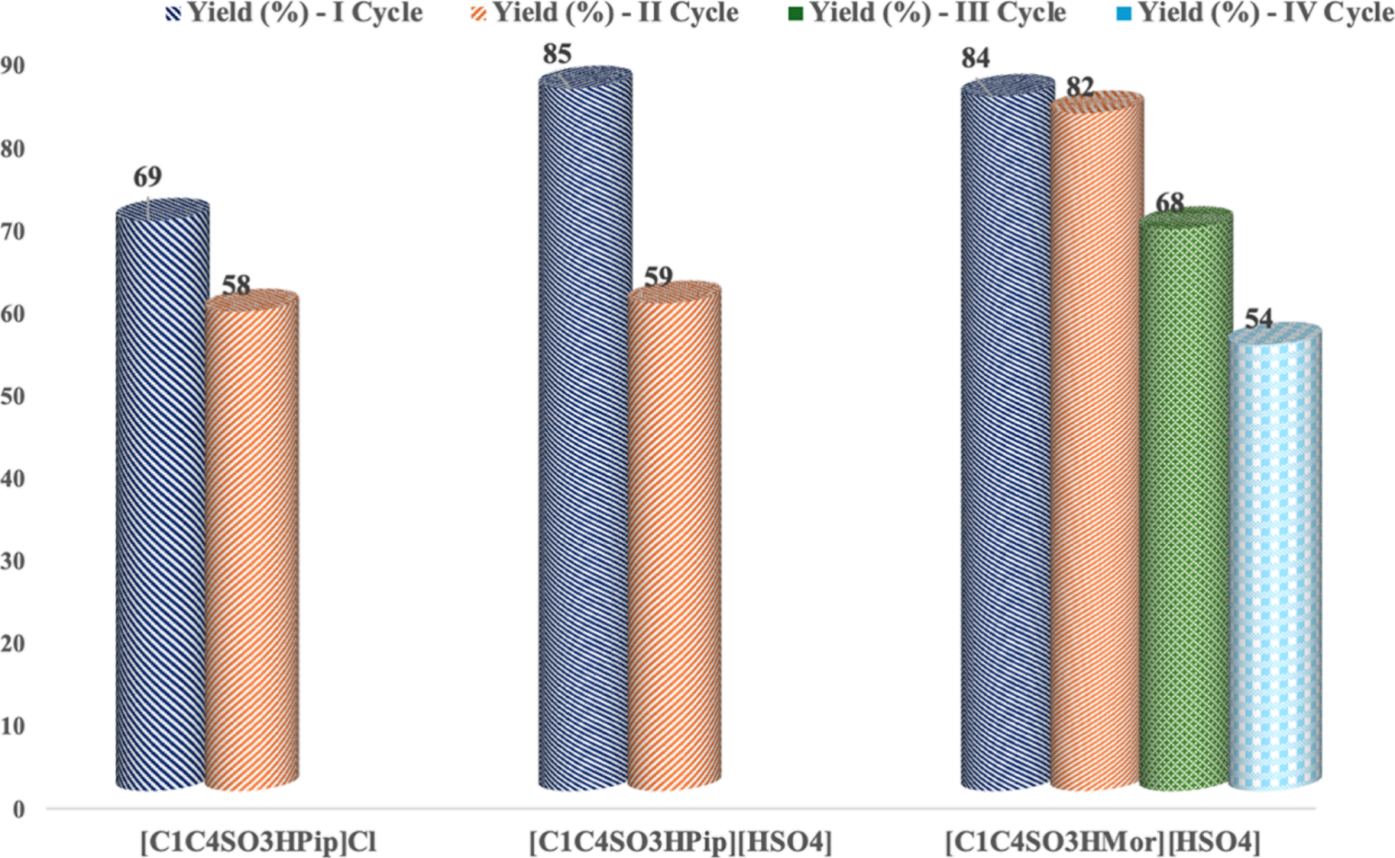
Yield values for the reuse of different catalysts in the ethanolysis of PLA at 100 °C and for 4 h under autogenous pressure (*n*_c_/*n*_RU_ = 0.15; *m*_PLA_/*m*_ROH_ = 0.25). In all cases, quantitative conversions were detected.

In all cases, catalyst reuse gave quantitative conversions. However, analysis of results obtained evidences how both cation and anion nature affected the catalyst reusability. Indeed, among the catalysts tested, [C_1_C_4_SO_3_HPip]Cl could be reused for at least two cycles without significant loss in yield. However, at the third cycle, no activity was detected. On the other hand, changing the anion from Cl^–^ to [HSO_4_^–^] decreased the catalyst reusability, as in the second cycle although the conversion stayed constant, a significant drop in yield from 85 down to 59% was detected. Differently, on going from [C_1_C_4_SO_3_HPip]­[HSO_4_] to [C_1_C_4_SO_3_HMor]­[HSO_4_], a significant improvement in catalyst performance was detected. Indeed, [C_1_C_4_SO_3_HMor]­[HSO_4_] could be reused for at least three cycles, and in the fourth one, although conversion stayed constant, a significant drop in yield was detected (54%). After the third cycle, we recorded the ^1^HNMR spectrum of the catalyst (Figure S3), evidencing the presence of impurities that probably account for its loss in activity.

Finally, the attempt to process a higher amount of polymer (5-fold) was carried out to verify the scalability of the proposed methodology. Under the best experimental conditions and using [C_1_C_4_SO_3_HMor]­[HSO_4_] as the catalyst, a quantitative conversion with a yield equal to 77% was obtained.

### Mechanistic Hypothesis

In light of the above results and having detected a certain dependence of the process performance from the nature of the ions constituting TSILs, a possible reaction mechanism was hypothesized (Scheme 3).

**Scheme 3:**
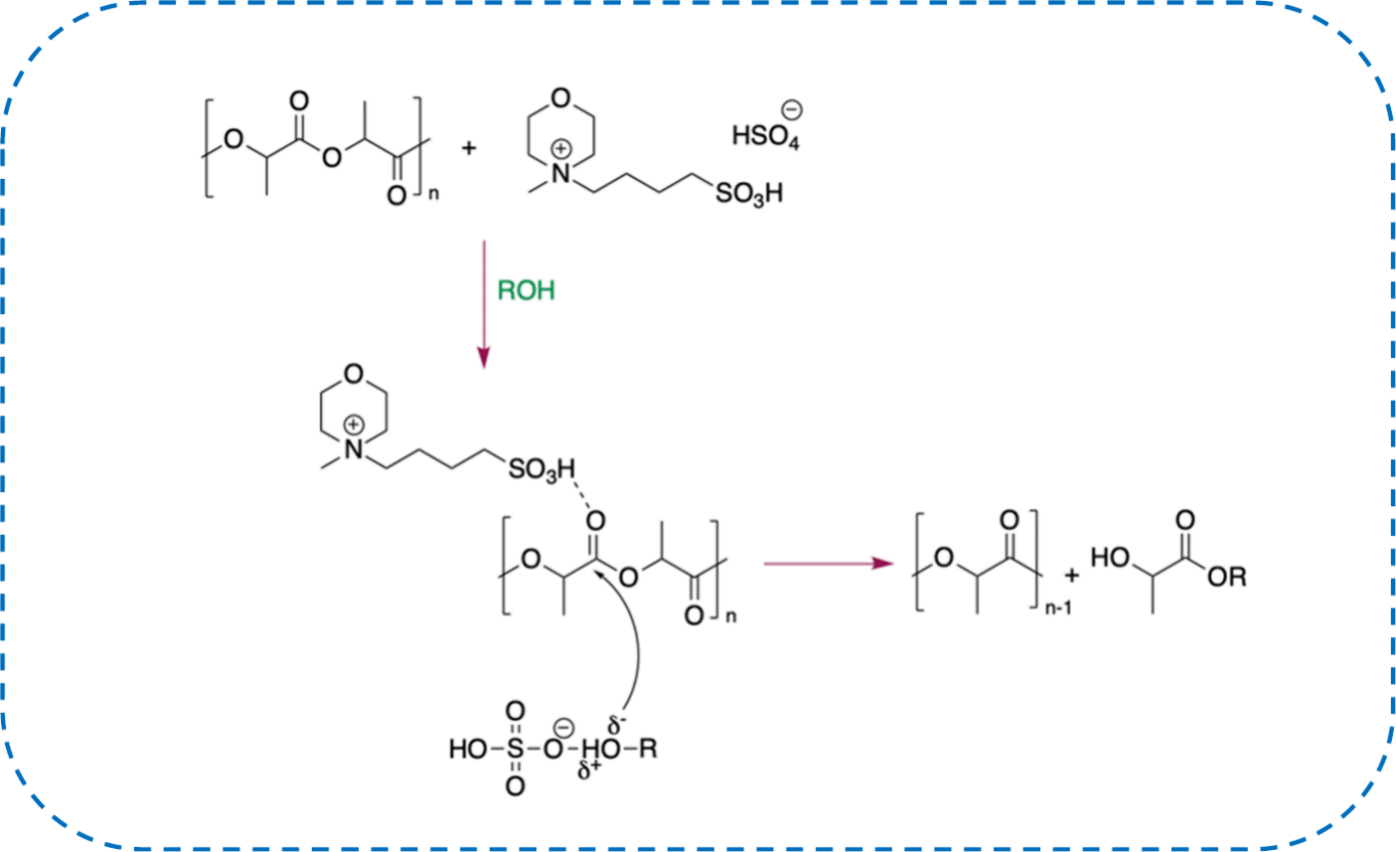
Possible Reaction Mechanism of the TSIL-Catalyzed Alcoholysis of PLA

According to the suggested hypothesis, both Cl^–^ and [HSO_4_^–^]-based TSILs could enhance the electrophilicity of the carbonyl group of the polyester through the acidic function on the cation, and this should favor the nucleophile addition. However, it is only in the presence of the [HSO_4_^–^] anion that a dual catalytic function could be exerted. Indeed, this anion, thanks to its amphiprotic nature, could at the same time act as a hydrogen bond donor toward the carbonyl group and a hydrogen bond acceptor toward the alcoholic hydroxyl group, determining the higher catalytic efficiency with respect to the corresponding chloride-based ILs.

### Sustainability Evaluation

Taking into consideration that the main aim of the work is upcycling of a waste to decrease the human footprint in the environment, a critical evaluation of the used reaction conditions is mandatory. To this aim, the guidelines suggested by Clark et al. through the holistic approach to Green Chemistry^39^ were considered. According to this approach, one of the main advantages of the methodology proposed derives from the possibility to obtain different lactate esters with yields going from satisfactory (52% for butyl lactate (BL)) up to excellent (84% for ethyl lactate (EL)).

The reaction temperature (100 °C) is in the range considered industrially admissible, and, thinking about the application of such methodology on a large scale, this could represent a benefit.

In addition, in the presence of both [C_1_C_4_SO_3_HPip]­[HSO_4_] and [C_1_C_4_SO_3_HMor]­[HSO_4_], quantitative conversions and yields higher than 80% were detected, perfectly accomplishing the target to award the process a green flag. On the other hand, among tested catalysts, one of the best performing catalysts, [C_1_C_4_SO_3_HMor]­[HSO_4_], bears the less toxic cationic head. Furthermore, the suggested MW-assisted procedure for catalyst precursors allowed their obtainment with yields ranging from 70 up to 98% in shorter reaction times with respect to the ones used under thermal conditions.

Finally, to better evaluate the performance of our procedure and to have insights on how to minimize the impact of the depolymerization process, comparison with data previously reported in the literature is mandatory. Analysis of previous data immediately underlines a significant difference. Indeed, we mainly focused our attention on the use of EtOH as the nucleophile, which can be obtained from ligno-cellulosic biomass, and reasoning in terms of the LCA approach, its production is of lower environmental cost with respect to MeOH, generally obtained from fossil fuels. In this case, comparison with the literature is quite difficult as reported cases mainly deal with methanolysis. In our conditions, although conversion of PLA was quantitative, also in the case of methanolysis, methyl lactate (ML) recovery was hampered by its higher solubility in the reaction mixture, containing the TSIL, with respect to the hexane phase. However, considering that our investigation reveals a decrease in yield in the presence of longer-chain alcohols, we are really confident that our best results in ethanolysis ([C_1_C_4_SO_3_Mor]­[HSO_4_] and ([C_1_C_4_SO_3_Pip]­[HSO_4_]) can be reasonably compared to previous methanolysis protocols. Furthermore, for a homogeneous comparison, the nature of the catalyst was kept constant, considering only IL-based catalysts. Data are reported in Table 3.

**Table 3:** Comparison with Data Previously Reported in the Literature about the Methanolysis of PLA

catalyst	*n*_c_/*n*_RU_	*m*_PLA_/*m*_ROH_	*T* (°C)	*t* (h)	yield (%)	catalyst reuse	reference
[C_1_C_4_SO_3_HMor][HSO_4_]	0.15	0.25	100	4	74		this work
[C_1_C_4_SO_3_HMor][HSO_4_]^a^	0.15	0.25	100	4	91	5	this work
[TMA]F	0.18	0.005	90	1	60	5	^40^
[bmim][OAc]	0.007	0.2	115	3	93	6	^28^
[bmim][AcO]-Zn(OAc)_2_	0.002	0.2	120	1	92	5	^29^
[bmim]Cl-FeCl_3_ (X_FeCl3_ = 0.67)	0.0025	0.2	110	3	88	6	^30^
[C_1_C_3_SO_3_HIm][HSO_4_]	0.005	0.2	115	3	87	6	^41^
[HDBU][AA]	0.05		100	5	91	6	^42^
[HDBU][Im]	0.002		100	2	96	6	^43^
[HTBD][odmGly]	0.02		70	3	71		^44^

a ROH: EtOH.

Analysis of data reported shows that our best catalyst exhibited, in this case, a good performance, with yield comparable or only slightly lower than the ones previously reported. However, in most of the previously reported cases, higher reaction temperatures were used. Furthermore, in most of the previously reported cases, imidazolium-based catalysts were used, and as widely recognized catalysts,^45^ these show higher cyto- and environmental toxicities with respect to morpholinium-based ones. Our best catalyst proved to have a good reusability, fully in line with the ones previously reported, and allowed scaling-up of the process without significant loss in performance. It is important to note that a meaningful and useful sustainability assessment should take into account several independent factors, such as reaction conditions, yield, and catalyst amount as well as its safety/toxicity and reusability. Based on this, our overall assessment is that this protocol is promising and can further advance the current search for more sustainable PLA depolymerization, especially in terms of mildness of reaction conditions, catalyst safety, and reusability.

## Conclusions

The presence of plastic waste in the environment is one of the pressing issues of modern society. To face up to this concern and identify methodologies aimed at upcycling this kind of waste, in this paper, a methodology directed to the obtainment of lactate esters from PLA is proposed. To the best of our knowledge, this is one of the few papers concerning the use of acidic TSILs to obtain lactate esters from PLA. In particular, the paper focused on the use of sulfonic acid-based ILs, which showed very high efficiency, also proving easy to handle with respect to the corresponding mineral acid. Furthermore, considering the wide range of industrial applicability of such kinds of substrates, different from most previously reported papers, we tested the general applicability of our protocol using alcohols having different alkyl chain lengths and viscosities.

On the grounds of guidelines derived from Green Chemistry Principles and the holistic approach to Green Chemistry, the whole process was designed aiming to minimize energy and materials consumption as well as cyto- and ecotoxicities of the reagents used. This is the reason why the use of aliphatic TSILs was preferred, and their synthesis was carried out under microwave irradiation, obtaining them in high yields and using significantly shorter reaction times with respect to the ones normally used under thermal heating. This allows significantly decreasing the energy demand of the process, improving the overall sustainability of the catalyst synthesis.

These catalysts enabled the obtainment of alkyl esters from PLA with quantitative conversions and yields ranging from 47 up to 88%. Their performance proved to be comparable or superior to the ones of the other IL-based catalysts previously reported with the same aim but bearing more toxic aromatic cations.

The best catalyst, [C_1_C_4_SO_3_HMor]­[HSO_4_], bearing the less toxic cationic head, could be reused for at least three cycles without a significant loss in performance and was successfully applied in the scale-up of the process.

The methodology proposed showed general applicability. Indeed, although it was optimized using EtOH, it allowed the obtainment of different lactate esters in good yields, generally considered products of industrial value and frequently obtained from fossil fuels. This makes our strategy a suitable way to reduce modern society's dependence on nonrenewable sources.

Finally, analysis of collected results under the best experimental conditions, in light of the holistic approach to Green Chemistry on laboratory scale, awards our protocol of green flags as far as conversion and yield values are concerned and allows us to consider the temperature of the process admissible on an industrial scale.

## Supplementary Material



## Notes

Characterization of the catalysts and lactate esters, tables for conversion and yield values for the alcoholysis of PLA under different experimental conditions, NMR spectra of catalysts and lactate esters, and FT-IR spectra of the catalysts and images corresponding to different steps of the methodology (PDF)
