# Supplementary material for: Valorization of Poly(lactic acid) to Lactate Esters Using Task-Specific Ionic Liquids
Source: ACS Sustain Chem Eng. 2025 Nov 21;13(48):20766–75. doi: 10.1021/acssuschemeng.5c08785 (PMC12691869; doi:10.1021/acssuschemeng.5c08785)
Supplement: Supplementary file 1 [file sc5c08785_si_001.pdf]

Supporting Information for

Valorization of Poly(lactic acid) to Lactate Esters using Task specific Ionic Liquids

*Salvatore Marullo, Martina Silaco, Francesca D'Anna**

Università degli Studi di Palermo, Dipartimento STEBICEF, Viale delle Scienze, Ed. 17, 90128 Palermo (Italy)

Email: francesca.danna@unipa.it

Pages: 20

Numbers of Tables: 5

Numbers of Figures: 5

Experimental Section Pag. S2

Table S1. Conversion and yield values for the ethanolysis of PLA, performed in the presence of [$C_1C_4SO_3$HPip][HSO_4] at 100 °C, for 2h under autogenous pressure, as function of catalyst loading and amount of solvent used. Pag. S5

Table S2. Conversion and yield values for the ethanolysis of PLA, performed in the presence of [$C_1C_4SO_3$HPip][HSO_4] or [$C_4C_4SO_3$HIm][HSO_4] at 100 °C, under autogenous pressure, as function of the time (n_c/n_{RU}: 0.15; m_{PLA}/m_{EtOH}: 0.25) Pag. S5

Table S3. Conversion and yield values for the ethanolysis of PLA, performed as a function of the catalyst, at 100 °C and for 4h, under autogenous pressure (n_c/n_{RU}: 0.15; m_{PLA}/m_{EtOH}: 0.25). Pag. S5

Table S4. Conversion and yield values for the alcoholysis of PLA, performed in the presence of [$C_1C_4SO_3$HMor][HSO_4], at 100 °C and for 4h, under autogenous pressure, as a function of the different nature of the nucleophile (n_c/n_{RU}: 0.15; m_{PLA}/m_{EtOH}: 0.25). Pag. S5

Table S5. Conversion and yield values for the reusing of different catalysts in the ethanolysis of PLA, performed in the at 100 °C and for 4h, under autogenous pressure (n_c/n_{RU}: 0.15; m_{PLA}/m_{EtOH}: 0.25). Pag. S5

Figure S1. NMR spectra of catalysts. Pag. S6-S14

Figure S2. NMR spectra of lactate esters. Pag. S15-S17

Figure S3. NMR spectrum of catalyst recovered after recycling. Pag. S17

Figure S4. FT-IR spectra of catalysts Pag. S18

Figure S5. Representative pictures of alcoholysis reaction Pag. S20

Experimental section

Characterization of zwitterionic compounds

[$C_{222}C_4SO_3)N$]

White solid; Yield: 65%; ^{1}H-NMR (400 MHz, D_2O): δ= 1.18 (t, J= 8.0 Hz, 9H), 1.73 (m, 4H), 2.88(t, J= 8.0 Hz, 2H), 3.20 ppm (q, J=8.0 Hz, 6H).

[$C_1C_4SO_3Mor$]

White solid; Yield: 86%; ^{1}H-NMR (400 MHz, D_2O): δ= 1.83-1.66 (m, 3 H), 1.94-1.83 (m, 2 H), 2.18-2.12 (m, 1 H), 2.93-2.89 (m, 2 H), 3.12 (s, 3 H), 3.28-3.25 (m, 1 H), 3.50-3.37 (m, 6 H) 4.54 ppm (t, J= 4 Hz, 1 H). ^{13}C-NMR (400 MHz, D_2O): δ=19.7, 21.2, 22.3, 50.3, 59.6, 60.4, 76.2 ppm.

[$C_1C_4SO_3Pip$]

White solid; Yield: 81%; ^{1}H-NMR (400 MHz, D_2O): δ= 1.56 (m, 2H), 1.79 (m, 8H), 2.89 (t, J = 8.0 Hz, 2H), 2.91 (s, 3H), 3.26 (m, 6H) ppm. ^{13}C-NMR (400 MHz, D_2O): δ= 19.5, 20.2,20.5, 21.2, 47.9, 50.0, 61.2, 75.9 ppm.

[$C_1C_4SO_3Im$]

White solid; Yield: 81%; ^{1}H-NMR (400 MHz, D_2O): δ= 0.82 (t, J = 8.0 Hz, 3H), 1.22 (quin, J = 8.0 Hz, 2H), 1.64 (m, 2H), 2.91 (s, 3H), 1.78 (quin, J = 8.0 Hz, 2H), 1.94 (quin, J = 8.0 Hz, 2H), 2.85 (m, 2H), 4.11 (t, J = 8.0 Hz, 2H), 4.16 (t, J = 8.0 Hz, 2H), 7.42 (d, J = 12.0 Hz, 2H), 8.73 (s, 1H) ppm.

Characterization of TSILs

[$C_{222}C_4SO_3HN$][Cl]

Pale yellow oil; Yield: 100%; ^{1}H-NMR (400 MHz, D_2O): δ=1.17 (t, J= 8.0 Hz, 9H), 1.72 (m, 4H), 2.86 (t, J = 8.0 Hz, 2H), 3.11 (t, J = 8.0 Hz, 2H), 3.19 (q, J = 8.0 Hz, 6H) ppm. ^{13}C-NMR (400 MHz, D_2O): δ=6.6, 19.9, 21.3, 50.0, 52.6, 56.0 ppm. FT-IR (liquid film): 3430, 1175, 1038 cm^{-1}.

[$C_{222}C_4SO_3HN$][HSO_4]

Pale yellow oil; Yield: 96%; ^{1}H-NMR (400 MHz, D_2O): δ= 1.13 (t, J= 8.0 Hz, 9H), 1.67 (m, 4H), 2.83 (t, J= 8.0 Hz, 2H), 3.07 (t, J = 8.0 Hz, 2H) 3.14 (q, J= 8.0 Hz, 6H), 3.59 (s, 1H) ppm. ^{13}C-NMR (400 MHz, D_2O): δ= 6.7, 19.9, 21.2, 45.0, 52.6, 55.5 ppm. FT-IR (liquid film): 3423, 1350, 1170, 1041 cm^{-1}.

[$C_1C_4SO_3HMor$][Cl]

Pale yellow oil; Yield: 92%; ^{1}H-NMR (400 MHz, D_2O): δ= 1.74 (quin, J = 8.0 Hz, 2H), 1.89 (m. 2H), 2.90 (t, J= 8.0 Hz, 2H), 3.10 (s, 3H), 3.43 (m, 4H), 3.96 (m, 4H) ppm. ^{13}C-NMR (400 MHz, D_2O): δ=19.9, 21.0, 22.3, 49.9, 59.7, 60.4, 76.3 ppm. FT-IR (liquid film): 3477, 1180, 1041 cm^{-1}.

[$C_1C_4SO_3HMor$][HSO_4]

Pale yellow oil; Yield: 93%; ^{1}H-NMR (400 MHz, D_2O): δ= 1.70 (t, J = 8.0 Hz, 2H), 1.83 (m, 1H), 2.86 (t, J = 8.0 Hz, 2H), 3.07 (s, 3H), 3.38 (m, 6H), 3.61 (s, 1H, exch.), 3.92 (m, 4H) ppm. ^{13}C-NMR (400 MHz, D_2O): δ=19.9, 21.1, 22.8, 49.9, 59.6, 60.4, 76.2 ppm. FT-IR (liquid film): 3340, 1352, 1170, 1042 cm^{-1}.

[$C_1C_4SO_3HPip$][Cl]

Pale yellow oil; Yield: 88%; ^{1}H-NMR (400 MHz, D_2O): δ= 1.55 (quin, J = 8.0 Hz, 2H), 1.86 (m, 8H), 2.88 (t, J = 8.0 Hz, 2H), 2.93 (s, 3H), 3.24 (m, 6H) ppm. ^{13}C-NMR (400 MHz, D_2O): δ= 19.5, 20.0, 20.5, 21.2, 50.0, 61.2, 62.6, 76.2 ppm. FT-IR (liquid film): 3435, 1179, 1040 cm^{-1}.

[$C_1C_4SO_3HPip$][HSO_4]

Pale yellow oil; Yield: 90%; ^{1}H-NMR (400 MHz, D_2O): δ= 1.50 (m, 2H), 1.73 (m, 8H), 2.83 (t, J = 8.0 Hz), 2.88 (s, 3H), 3.19 (m, 6H), 3.59 (s, 1H exch.) ppm. FT-IR (liquid film): 3392, 1355, 1169, 1041 cm^{-1}.

[$C_4C_4SO_3HIm$][Cl]

Colorless oil; Yield: 97%; ^{1}H-NMR (400 MHz, D_2O): δ= 1.01 (t, J = 8.0 Hz, 3H), 1.40 (sext, J = 8.0 Hz, 2H), 1.83 (m, 2H) 1.95 (quin, J = 8.0 Hz, 2H), 2.12 (quin, J = 8.0 Hz, 2H), 3.03 (t, J = 8.0 Hz, 2H), 4.29 (t, J = 8.0 Hz, 2H), 4.34 (t, J = 8.0 Hz, 2H), 7.62 (s, 2H), 8.91 (s, 1H) ppm. FT-IR (liquid film): 3444, 1649, 1175, 1038 cm^{-1}.

[$C_4C_4SO_3HIm$][HSO_4]

Colorless oil; Yield: 99%; ^{1}H-NMR (400 MHz, D_2O): δ= 0.77 (t, J = 8.0 Hz, 3H), 1.16 (sext, J = 8.0 Hz, 2H), 1.58 (m, 2H) 1.71 (quin, J = 8.0 Hz, 2H), 1.89 (quin, J = 8.0 Hz, 2H), 2.80 (t, J = 8.0 Hz, 2H), 3.59 (s, 1H), 4.06 (t, J = 8.0 Hz, 2H), 4.11 (t, J = 8.0 Hz, 2H), 7.37 (s, 2H), 8.66 (s, 1H) ppm.

Characterization of lactate esters

Methyl lactate

Colorless oil; ^{1}H-NMR (400 MHz, $CDCl_3$): δ= 1.34 (d, J = 8.0 Hz, 3H), 3.69 (s, 3H), 4.23 (q, J = 8.0 Hz, 1H) ppm.

Ethyl lactate

Colorless oil; ^{1}H-NMR (400 MHz, $CDCl_3$): δ= 1.28 (t, J = 8.0 Hz, 3H), 1.40 (d, J = 8.0 Hz, 3H), 3.70 (s, 1H), 4.23 (m, 3H) ppm.

Propyl lactate

Colorless oil; ^{1}H-NMR (400 MHz, $CDCl_3$): δ= 0.95 (t, J = 8.0 Hz, 3H), 1.40 (d, J = 8.0 Hz, 3H), 1.54 (m, 2H), 3.28 (s, 1H), 4.17 (m, 2H), 5.19 (m, 1H) ppm.

Isopropyl lactate

Colorless oil; ^{1}H-NMR (400 MHz, $CDCl_3$): δ=1.25 (m, 3H), 1.51 (m, 3H), 1.59 (m, 3H), 4.40 (m, 1H), 5.10 (m, 1H), 5.18 (m, 1H) ppm.

Butyl lactate

Colorless oil; ^{1}H-NMR (400 MHz, $CDCl_3$): δ= 0.95 (t, J = 8.0 Hz, 3H), 1.42 (d, J = 8.0 Hz, 3H), 1.53 (m, 2H), 1.66 (quin, J = 8.0 Hz, 2H), 2.71 (s, 1H), 4.23 (m, 2H), 5.19 (m, 1H) ppm.

Table S1. Conversion and yield values for the ethanolysis of PLA, performed in the presence of [$C_1C_4SO_3HPip$][HSO_4] at 100 °C, for 2h under autogenous pressure, as function of catalyst loading and amount of solvent used.

Catalyst loading (nc/cRU)	*PLA/EtOH (w/w)*	*Conversion (%)*[a]	*Yield (%)*[a]
0.10	1:4	100	35
0.10	1:6	100	40
0.15	1:6	100	51

[a]Values were reproducible within ± 3%.

Table S2. Conversion and yield values for the ethanolysis of PLA, performed in the presence of [$C_1C_4SO_3HPip$][HSO_4] or [$C_4C_4SO_3HIm$][HSO_4] at 100 °C, under autogenous pressure, as function of the time (n_c/n_{RU}: 0.15; m_{PLA}/m_{EtOH}: 0.25)

	**[$C_1C_4SO_3HPip$][HSO_4]**		**[$C_4C_4SO_3HIm$][HSO_4]**	
Reaction Time (h)	*Conversion (%)*[a]	*Yield (%)*[a]	*Conversion (%)*[a]	*Yield (%)*[a]
2	69	51	28	5
3	100	70	100	45
4	100	87	100	85

[a]Values were reproducible within ± 3%.

Table S3. Conversion and yield values for the ethanolysis of PLA, performed as a function of the catalyst, at 100 °C and for 4h, under autogenous pressure (n_c/n_{RU}: 0.15; m_{PLA}/m_{EtOH}: 0.25).

Catalyst	*Conversion (%)*[a]	*Yield (%)*[a]
[$C_4C_4SO_3HIm$][HSO_4]	100	85
[$C1C_4SO_3HMor$][HSO_4]	100	84
[$C1C_4SO_3HPip$][HSO_4]	100	88
[$(C_2)_3C_4SO_3HN$][HSO_4]	100	81
[$C1C_4SO_3HMor$][Cl]	100	64
[$C1C_4SO_3HPip$][Cl]	100	69
[$(C_2)_3C_4SO_3HN$][Cl]	100	47

[a]Values were reproducible within ± 3%.

Table S4. Conversion and yield values for the ethanolysis of PLA, performed in the presence of [$C_1C_4SO_3HMor$][HSO_4], at 100 °C and for 4h, under autogenous pressure, as a function of the different nature of the nucleophile (n_c/n_{RU}: 0.15; m_{PLA}/m_{EtOH}: 0.25).

Nucleophile	*Conversion (%)*[a]	*Yield (%)*[a]
MeOH	100	26
EtOH	100	84
PrOH	83	74
2-PrOH	100	57
BuOH	100	52

[a]Values were reproducible within ± 3%.

Table S5. Conversion and yield values for the reusing of different catalysts in the ethanolysis of PLA, performed in the at 100 °C and for 4h, under autogenous pressure (n_c/n_{RU}: 0.15; m_{PLA}/m_{EtOH}: 0.25).

	**[$C_1C_4SO3HPip$]Cl**		**[$C_1C_4SO_3HPip$][HSO_4]**		**[$C_1C_4SO_3HMor$][HSO_4]**	
	Conversion (%)[a]	*Yield (%)*[a]	*Conversion (%)*[a]	*Yield (%)*[a]	*Conversion (%)*[a]	*Yield (%)*[a]
I Cycle	100	69	100	85	100	84
II Cycle	100	58	100	59	100	82
III Cycle	-	-	-	-	100	68
IV Cycle	-	-	-	-	100	54

[a]Values were reproducible within ± 3%.

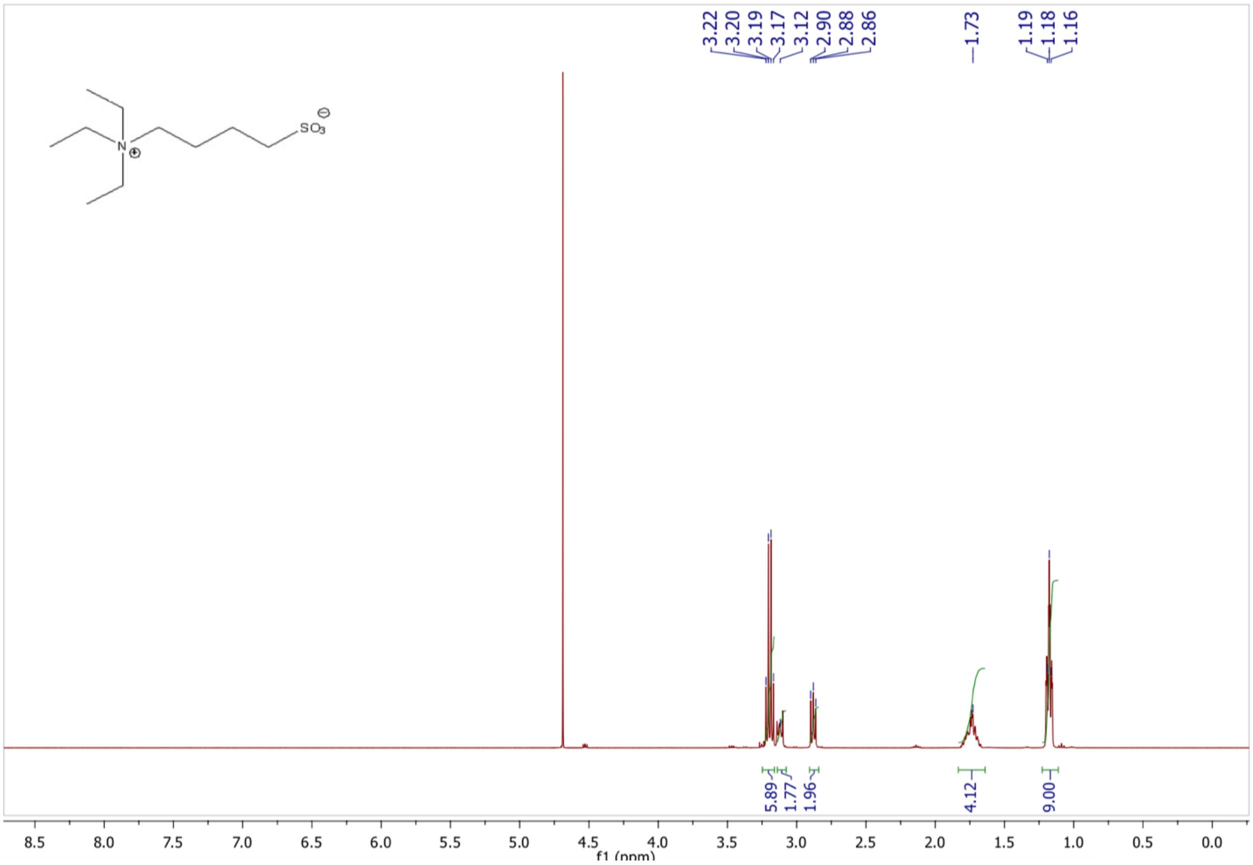

SO3
N
3.22
3.20
3.19
3.17
3.12
2.90
2.88
2.86
1.73
1.19
1.18
1.16
5.89
1.77
1.96
4.12
9.00
8.5 8.0 7.5 7.0 6.5 6.0 5.5 5.0 4.5 4.0 3.5 3.0 2.5 2.0 1.5 1.0 0.5 0.0
f1 (ppm)

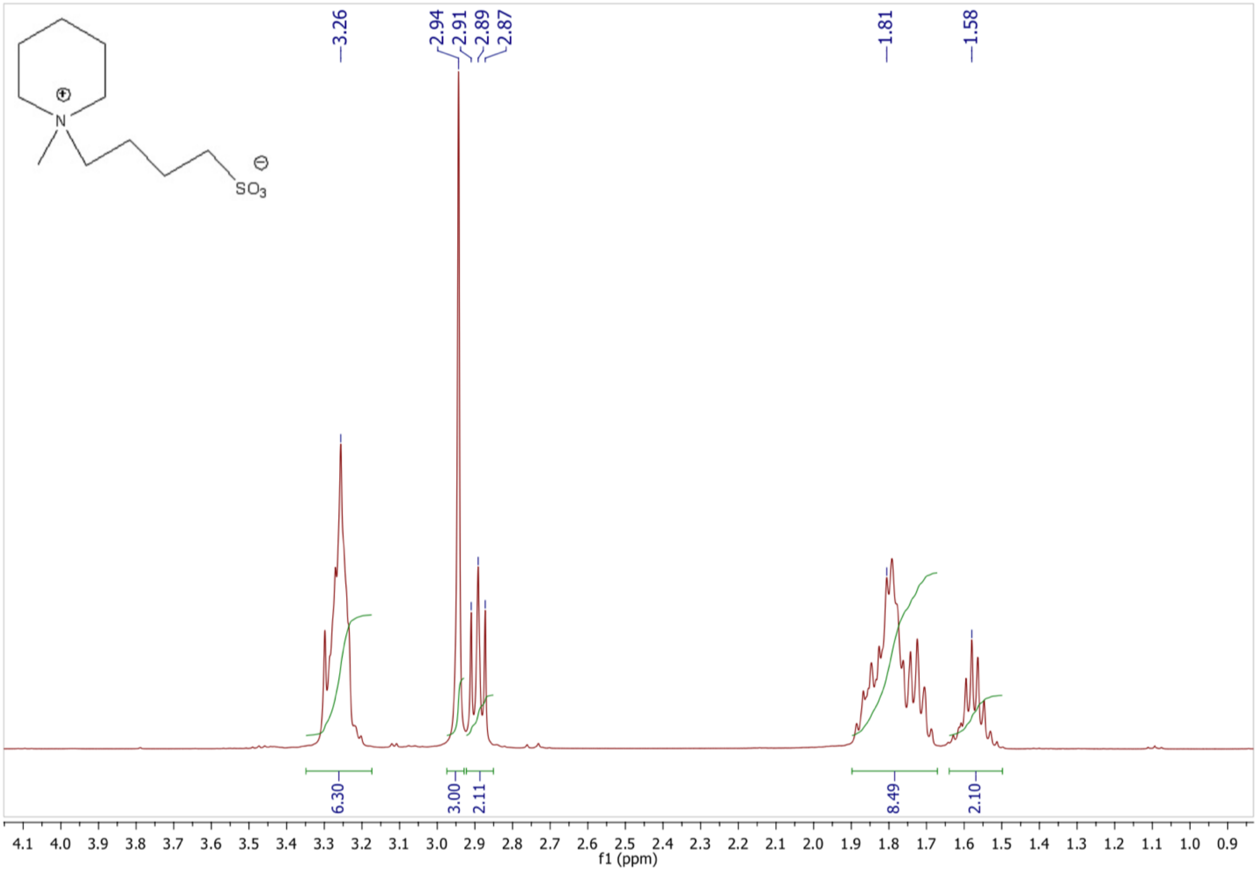

N
SO3
3.26
2.94
2.91
2.89
2.87
1.81
1.58
6.30
3.00
2.11
8.49
2.10
4.1 4.0 3.9 3.8 3.7 3.6 3.5 3.4 3.3 3.2 3.1 3.0 2.9 2.8 2.7 2.6 2.5 2.4 2.3 2.2 2.1 2.0 1.9 1.8 1.7 1.6 1.5 1.4 1.3 1.2 1.1 1.0 0.9
f1 (ppm)

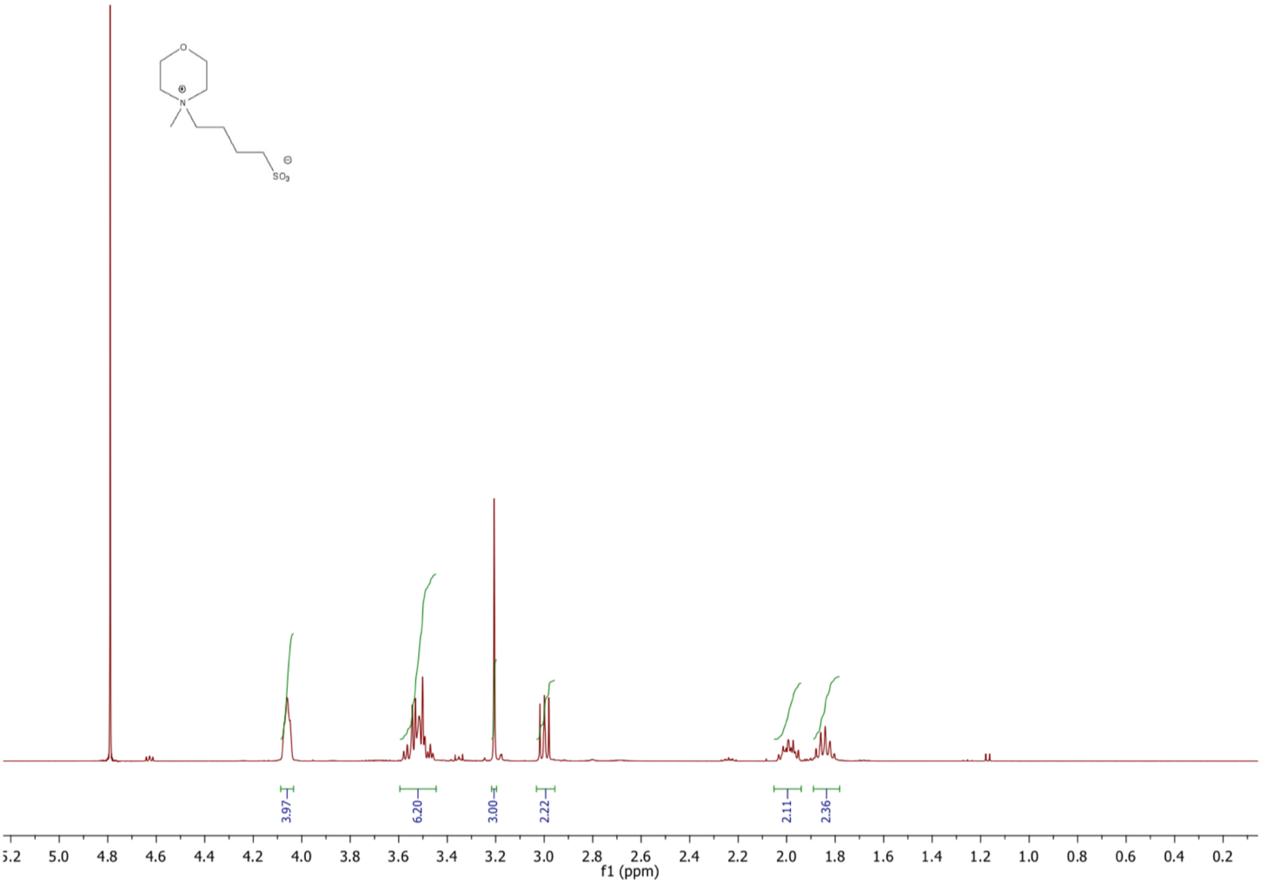

O
N
SO3
3.97
6.20
3.00
2.22
2.11
2.36
5.2 5.0 4.8 4.6 4.4 4.2 4.0 3.8 3.6 3.4 3.2 3.0 2.8 2.6 2.4 2.2 2.0 1.8 1.6 1.4 1.2 1.0 0.8 0.6 0.4 0.2
f1 (ppm)

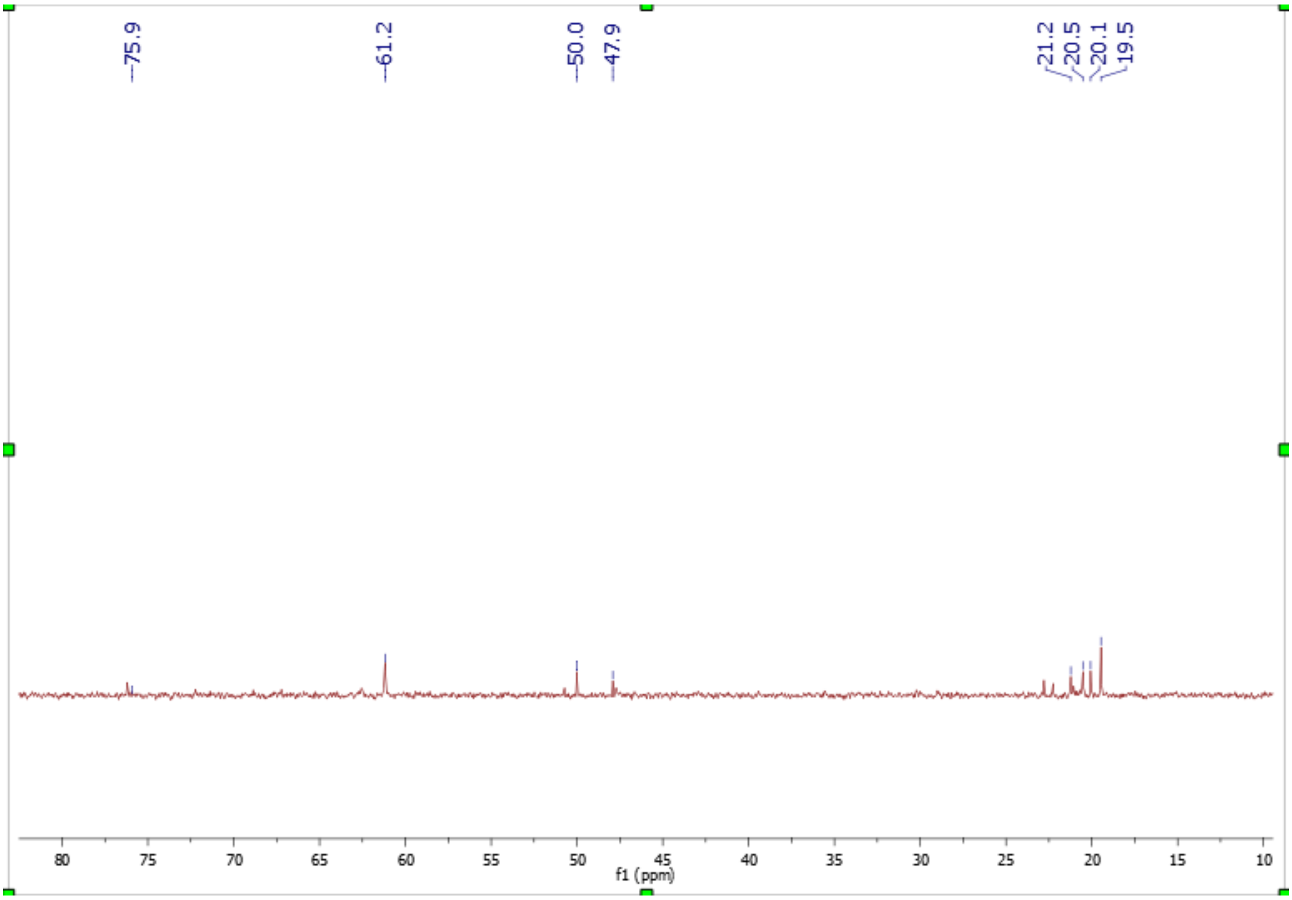

75.9
61.2
50.0
47.9
21.2
20.5
20.1
19.5
80 75 70 65 60 55 50 45 40 35 30 25 20 15 10
f1 (ppm)

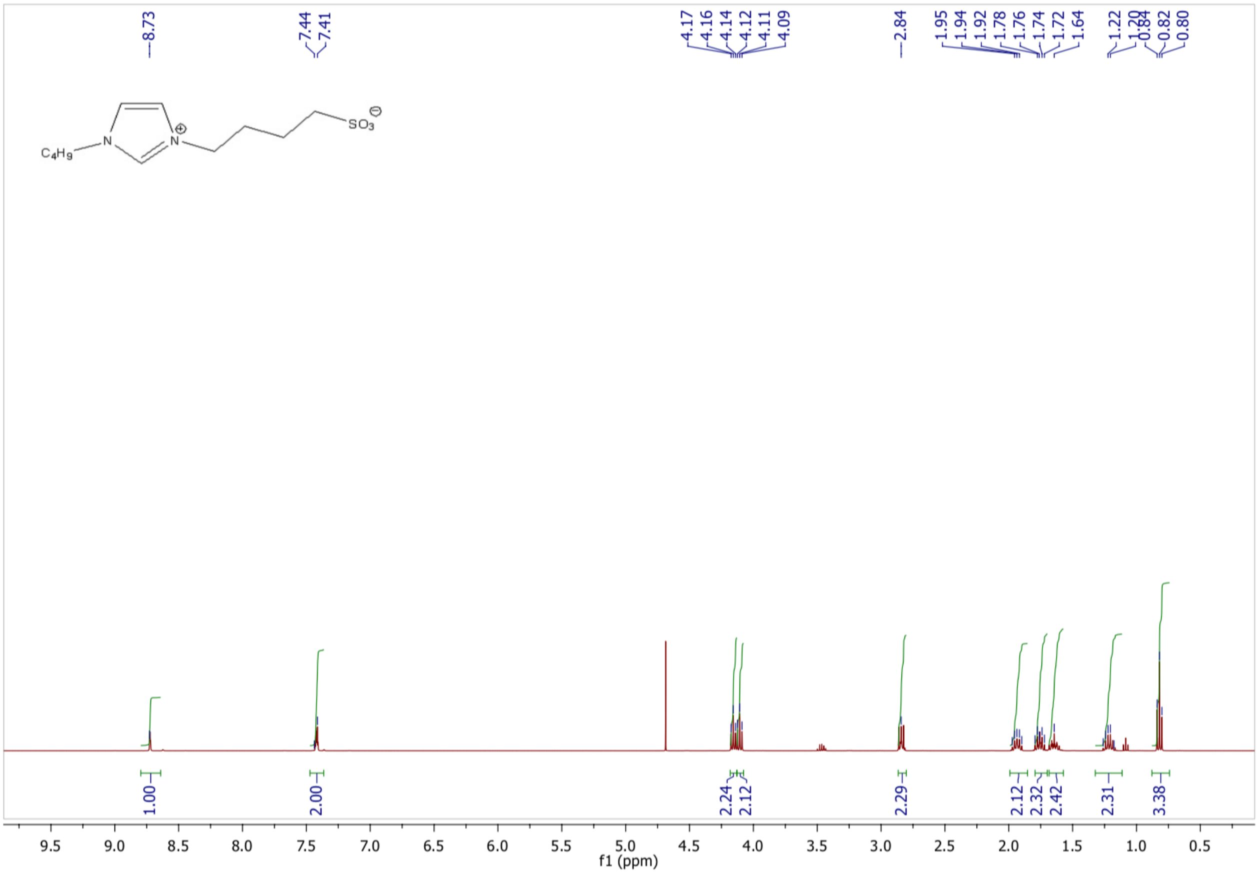
8.73
7.44
7.41
4.17
4.16
4.14
4.12
4.11
4.09
2.84
1.95
1.94
1.92
1.78
1.76
1.74
1.72
1.64
1.22
0.82
0.80
C4H9
N
N
SO3
1.00
2.00
2.24
2.12
2.29
2.12
2.32
2.42
2.31
3.38
9.5
9.0
8.5
8.0
7.5
7.0
6.5
6.0
5.5
5.0
4.5
4.0
3.5
3.0
2.5
2.0
1.5
1.0
0.5
f1 (ppm)

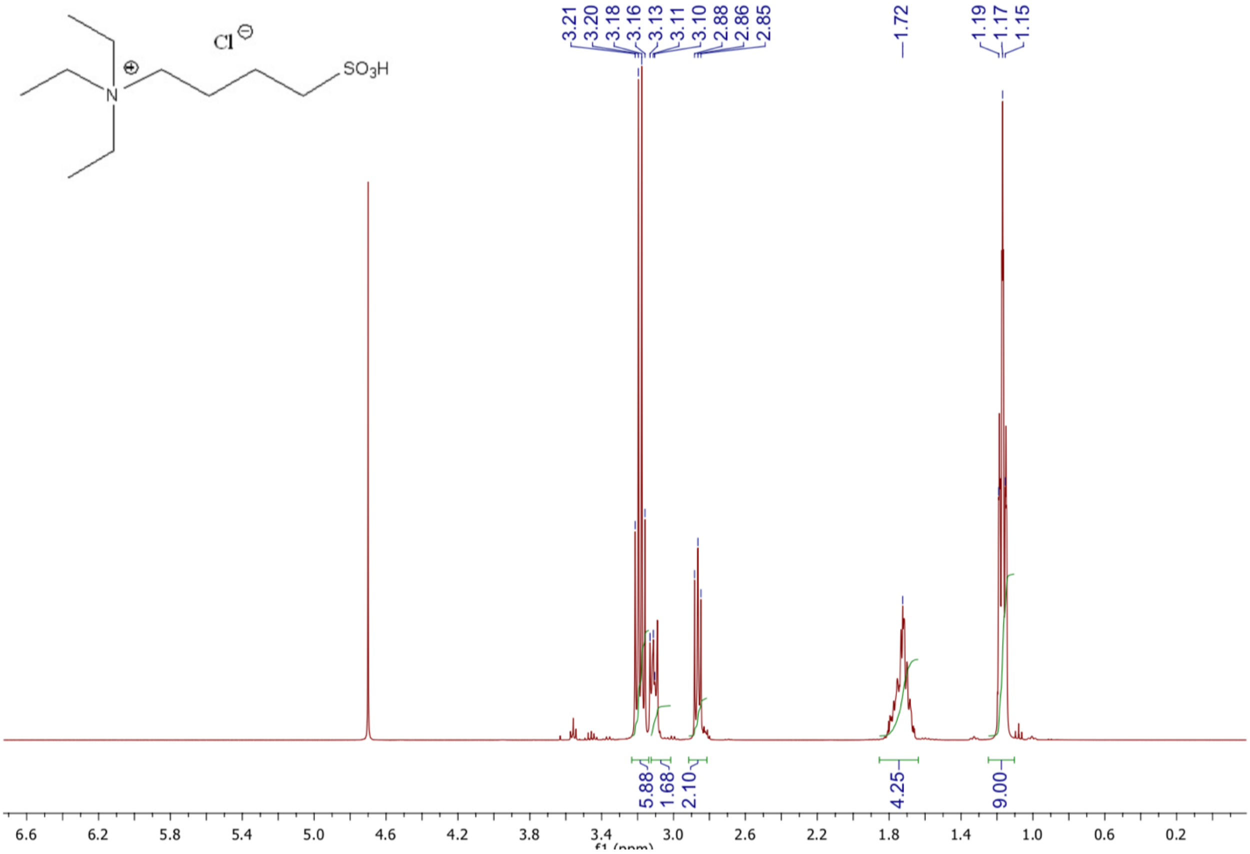
Cl⊖
⊕
N
SO3H
3.21
3.20
3.18
3.16
3.13
3.11
3.10
2.88
2.86
2.85
1.72
1.19
1.17
1.15
5.88
1.68
2.10
4.25
9.00
6.6 6.2 5.8 5.4 5.0 4.6 4.2 3.8 3.4 3.0 2.6 2.2 1.8 1.4 1.0 0.6 0.2

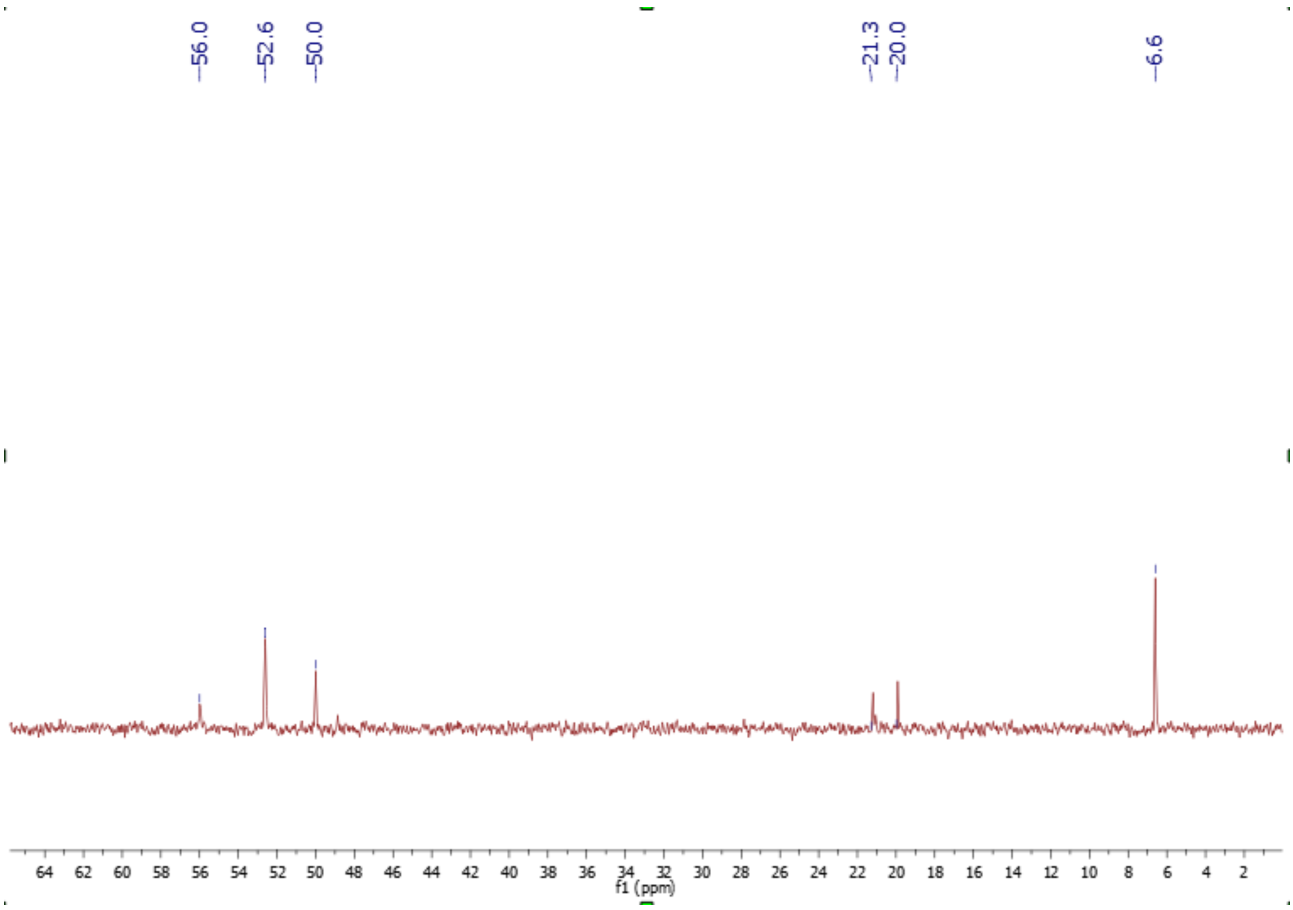
56.0
52.6
50.0
21.3
20.0
6.6
64 62 60 58 56 54 52 50 48 46 44 42 40 38 36 34 32 30 28 26 24 22 20 18 16 14 12 10 8 6 4 2
f1 (ppm)

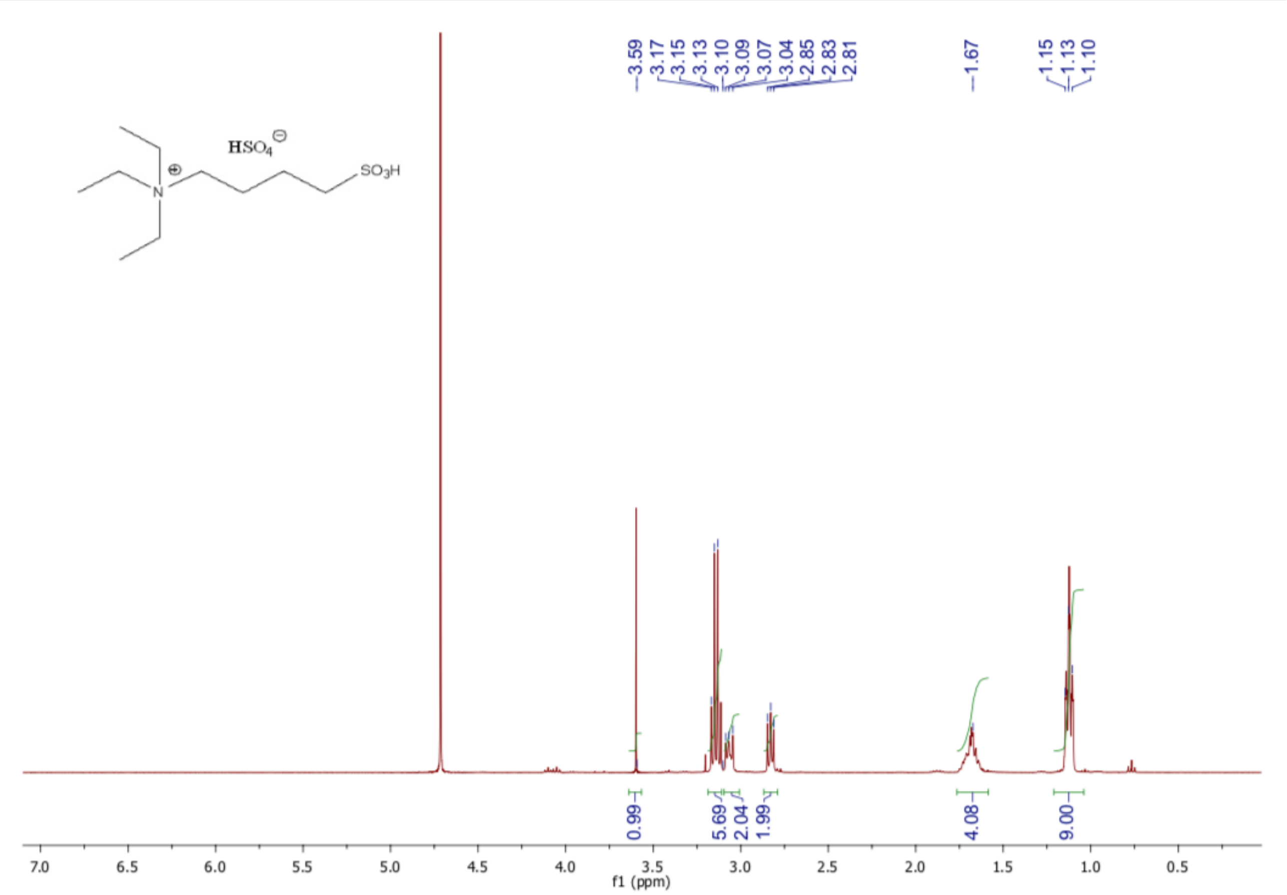
HSO4
SO3H
N
3.59
3.17
3.15
3.13
3.10
3.09
3.07
3.04
2.85
2.83
2.81
1.67
1.15
1.13
1.10
0.99
5.69
2.04
1.99
4.08
9.00
7.0 6.5 6.0 5.5 5.0 4.5 4.0 3.5 3.0 2.5 2.0 1.5 1.0 0.5
f1 (ppm)

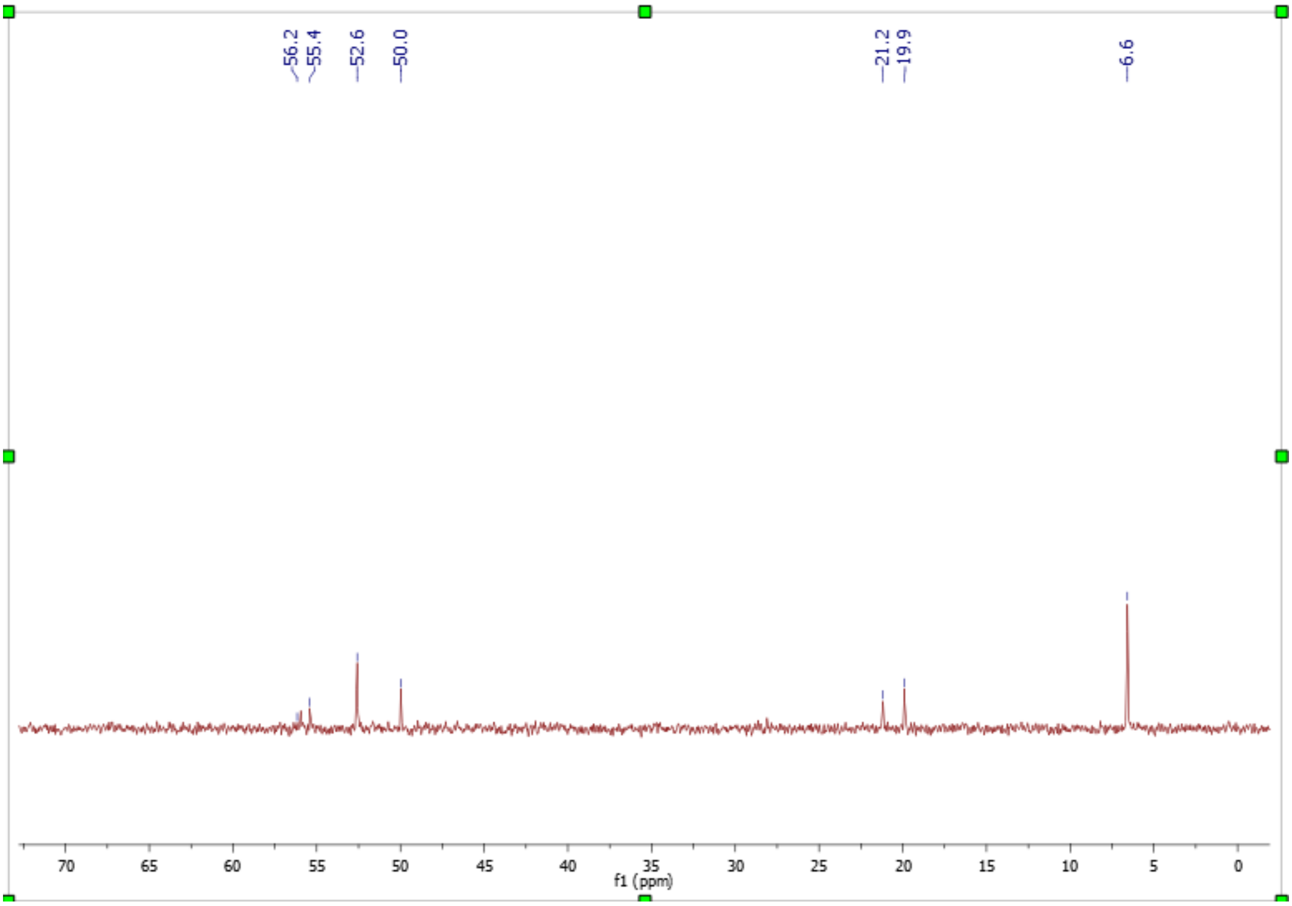
56.2
55.4
52.6
50.0
21.2
19.9
6.6
70 65 60 55 50 45 40 35 30 25 20 15 10 5 0
f1 (ppm)

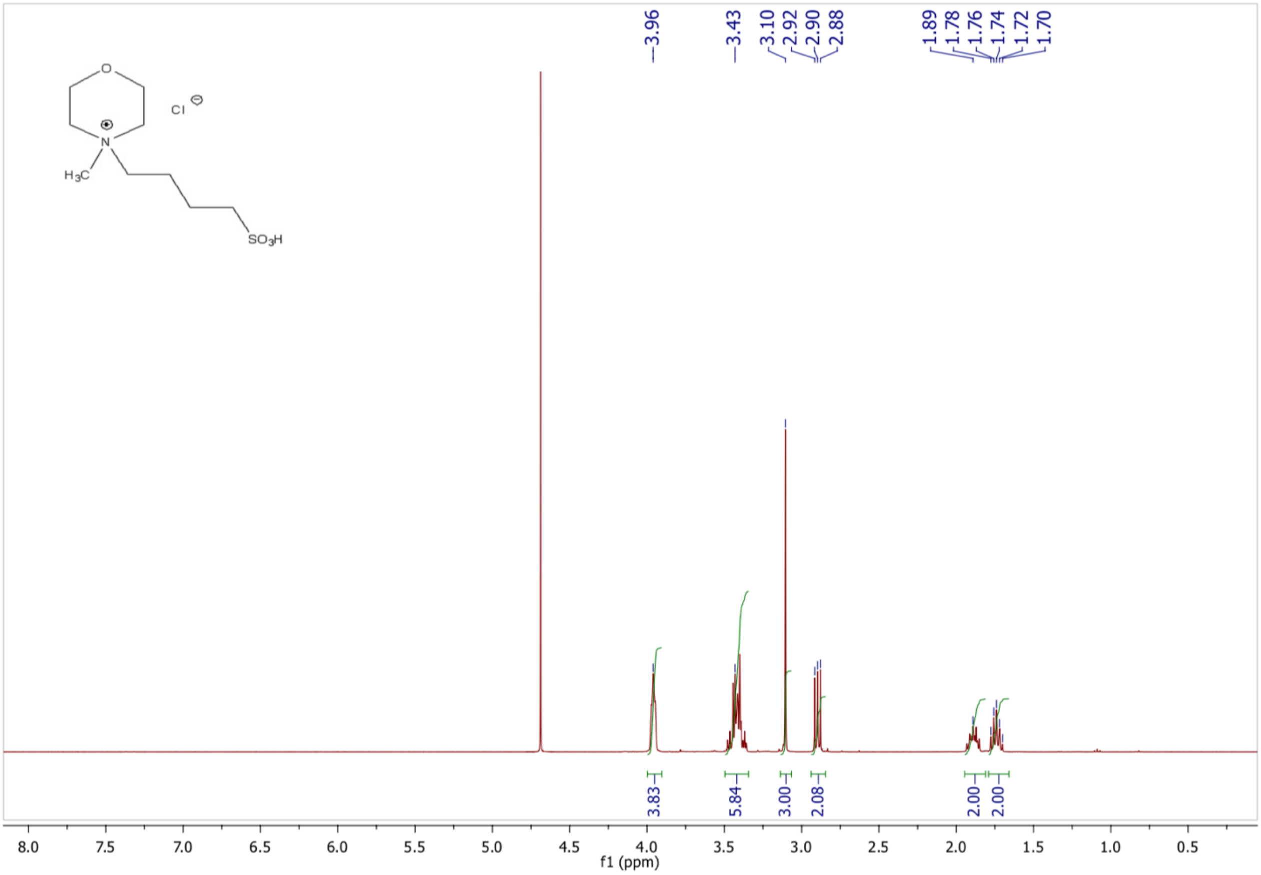
Cl
H3C
N
O
SO3H
3.96
3.43
3.10
2.92
2.90
2.88
1.89
1.78
1.76
1.74
1.72
1.70
3.83
5.84
3.00
2.08
2.00
2.00
8.0 7.5 7.0 6.5 6.0 5.5 5.0 4.5 4.0 3.5 3.0 2.5 2.0 1.5 1.0 0.5
f1 (ppm)

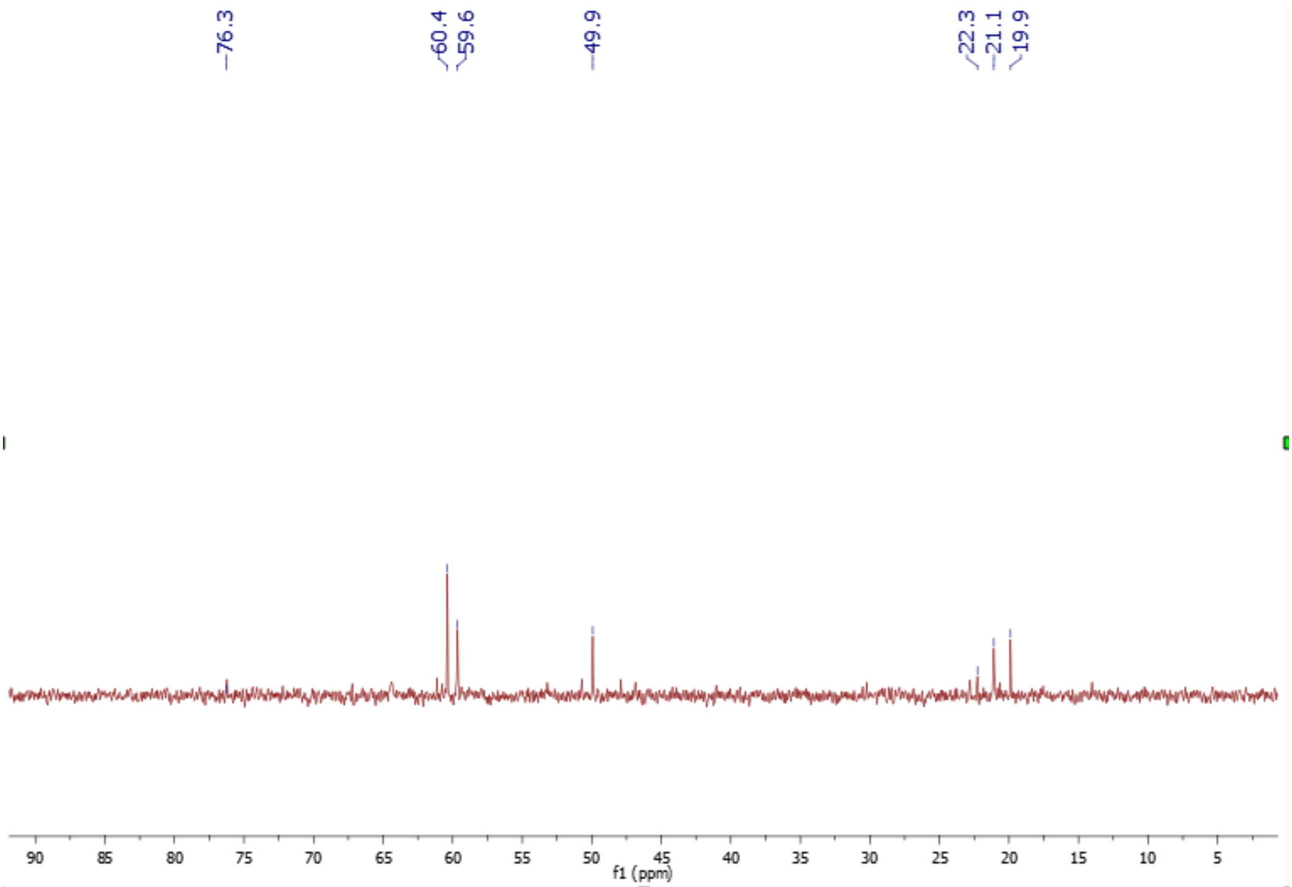
76.3
60.4
59.6
49.9
22.3
21.1
19.9
90 85 80 75 70 65 60 55 50 45 40 35 30 25 20 15 10 5
f1 (ppm)

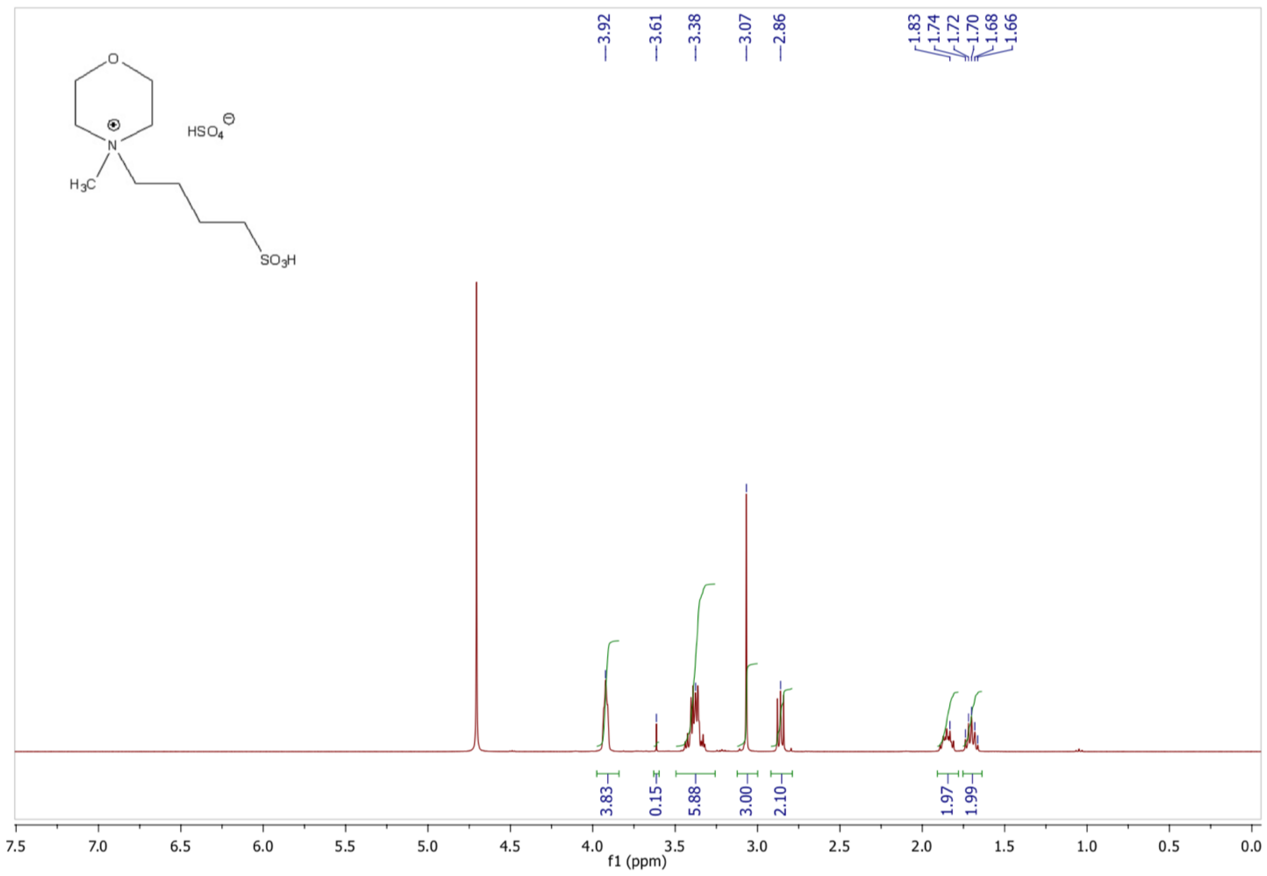
HSO4
H3C
N
O
SO3H
3.92
3.61
3.38
3.07
2.86
1.83
1.74
1.72
1.70
1.68
1.66
3.83
0.15
5.88
3.00
2.10
1.97
1.99
f1 (ppm)

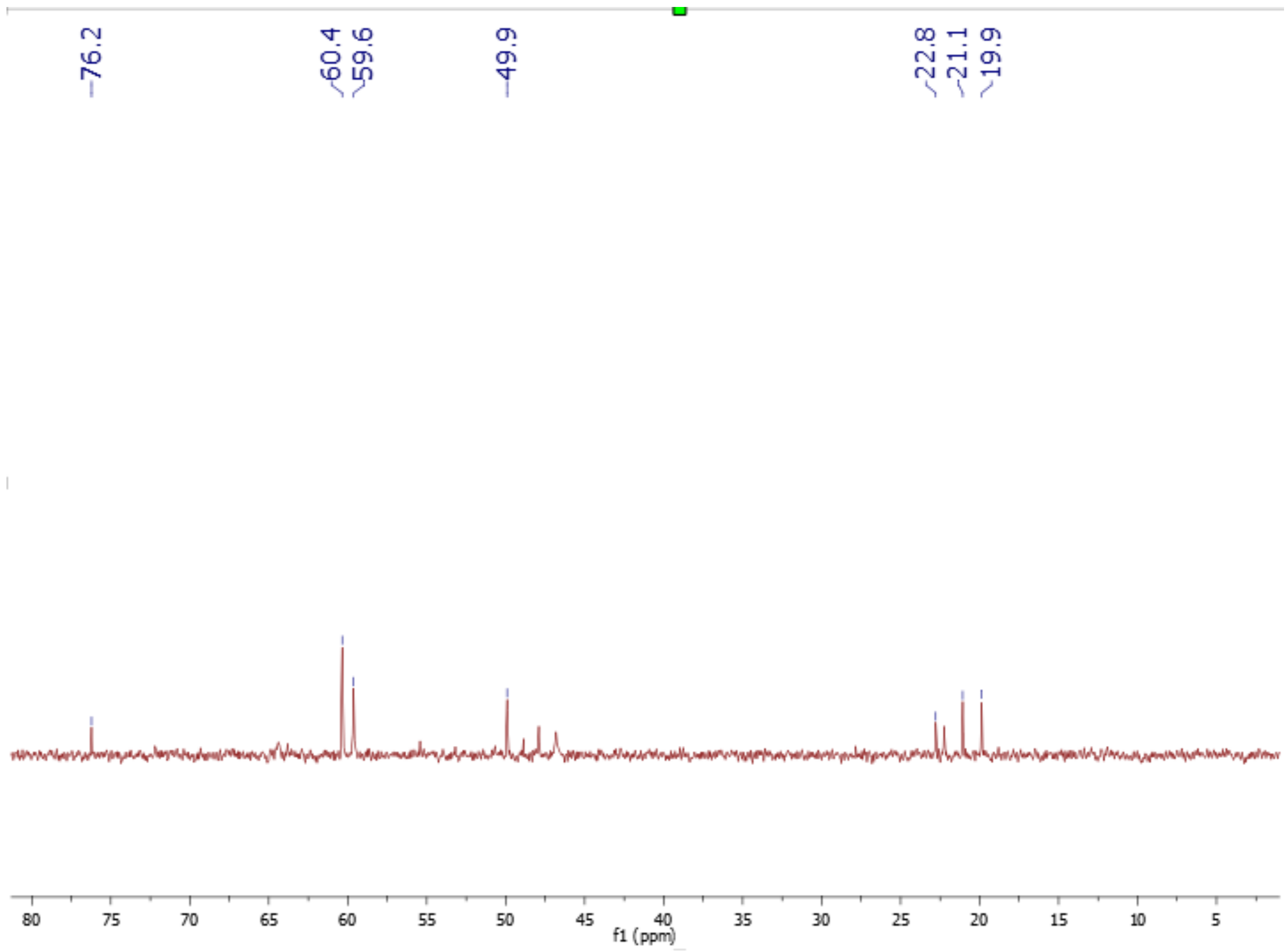
76.2
60.4
59.6
49.9
22.8
21.1
19.9
f1 (ppm)

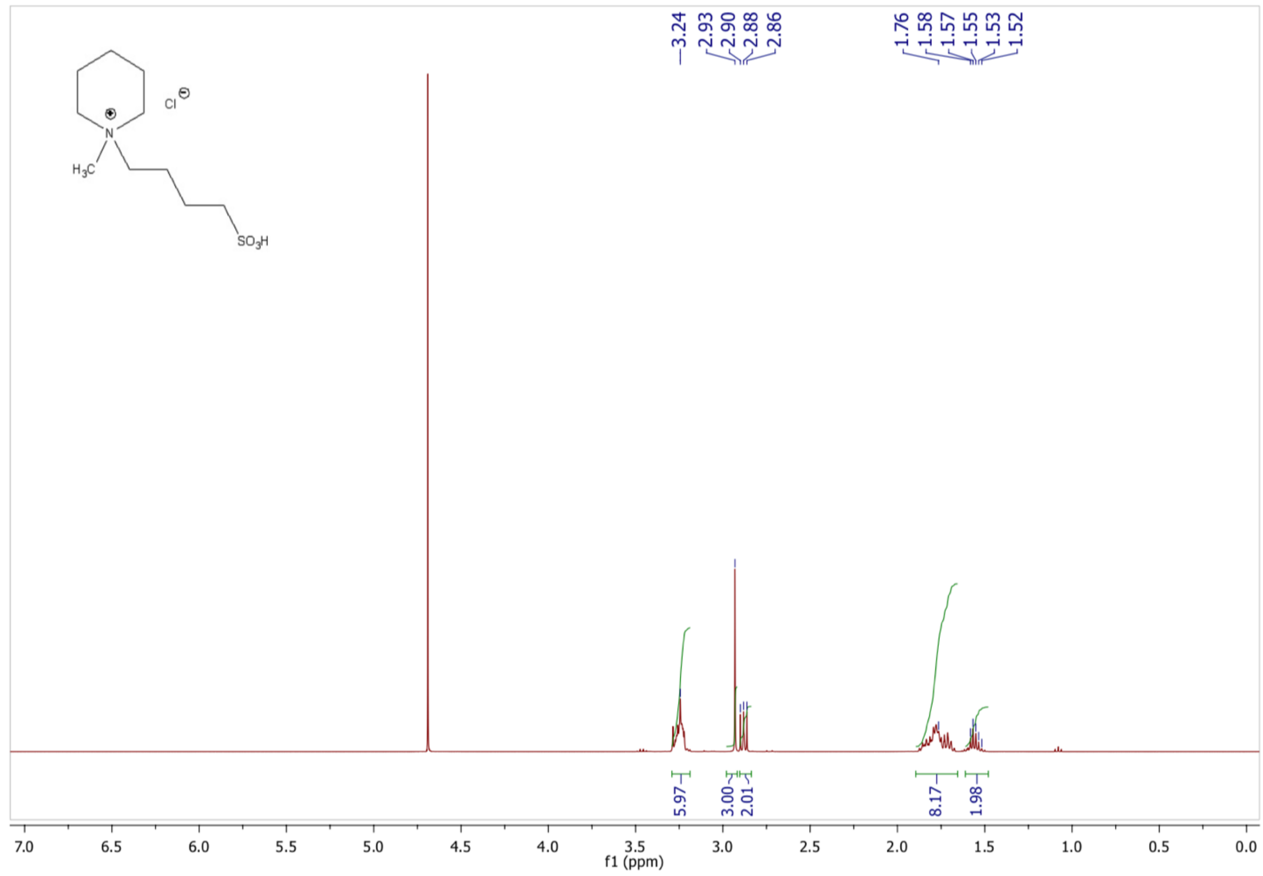

Cl
H3C
N
SO3H
3.24
2.93
2.90
2.88
2.86
1.76
1.58
1.57
1.55
1.53
1.52
5.97
3.00
2.01
8.17
1.98
f1 (ppm)

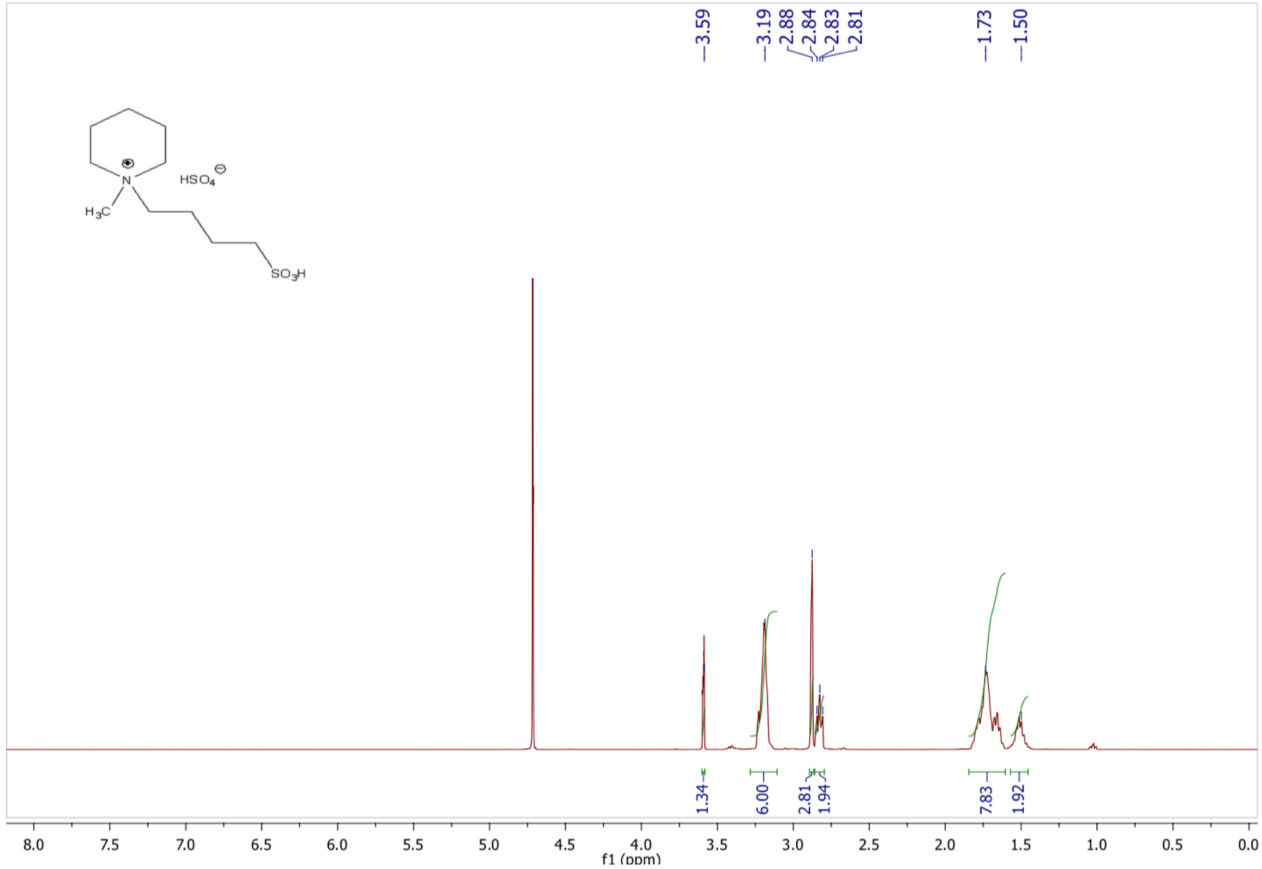

HSO4
H3C
N
SO3H
3.59
3.19
2.88
2.84
2.83
2.81
1.73
1.50
1.34
6.00
2.81
1.94
7.83
1.92
f1 (ppm)

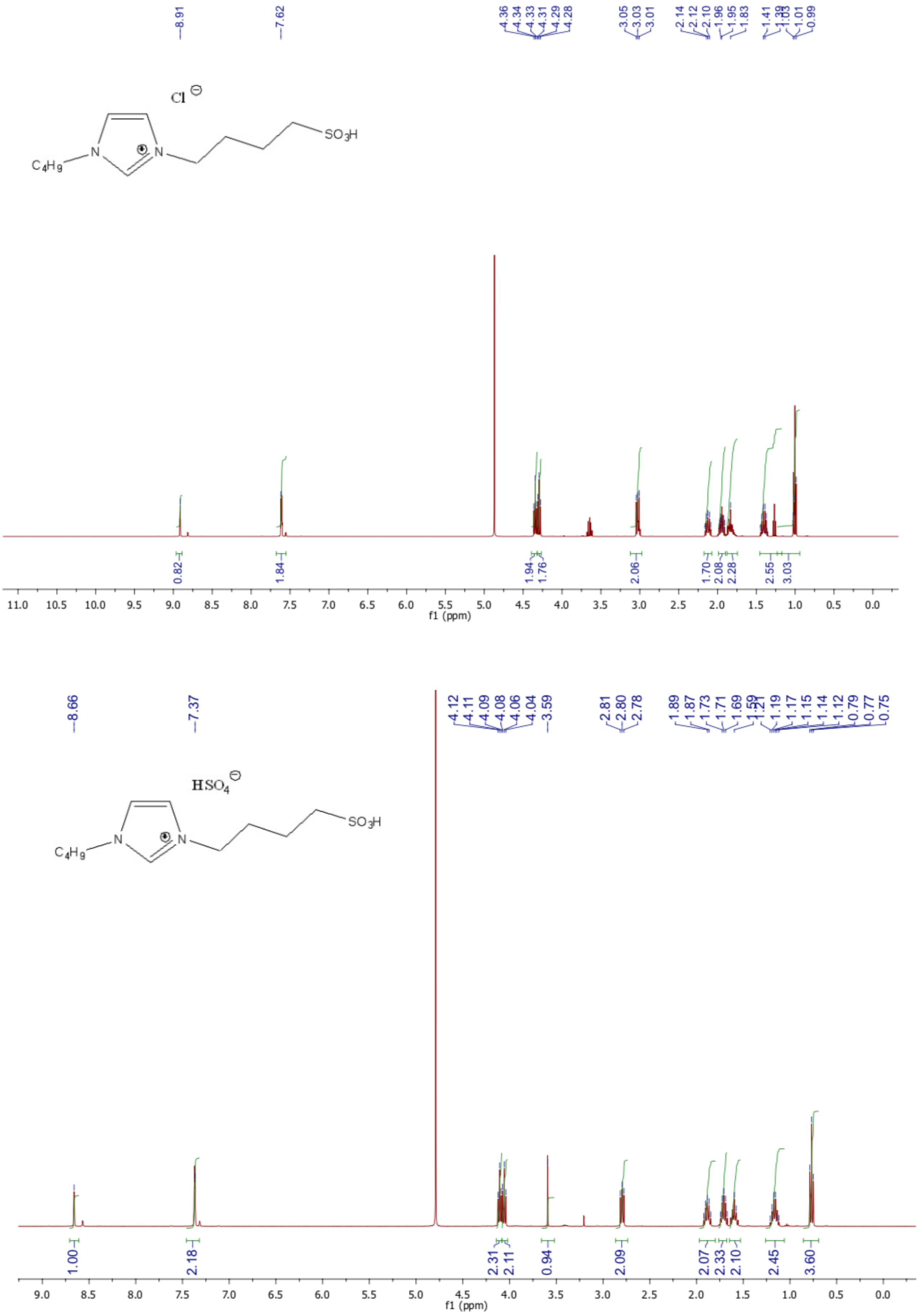

Figure S1. NMR spectra of catalysts.

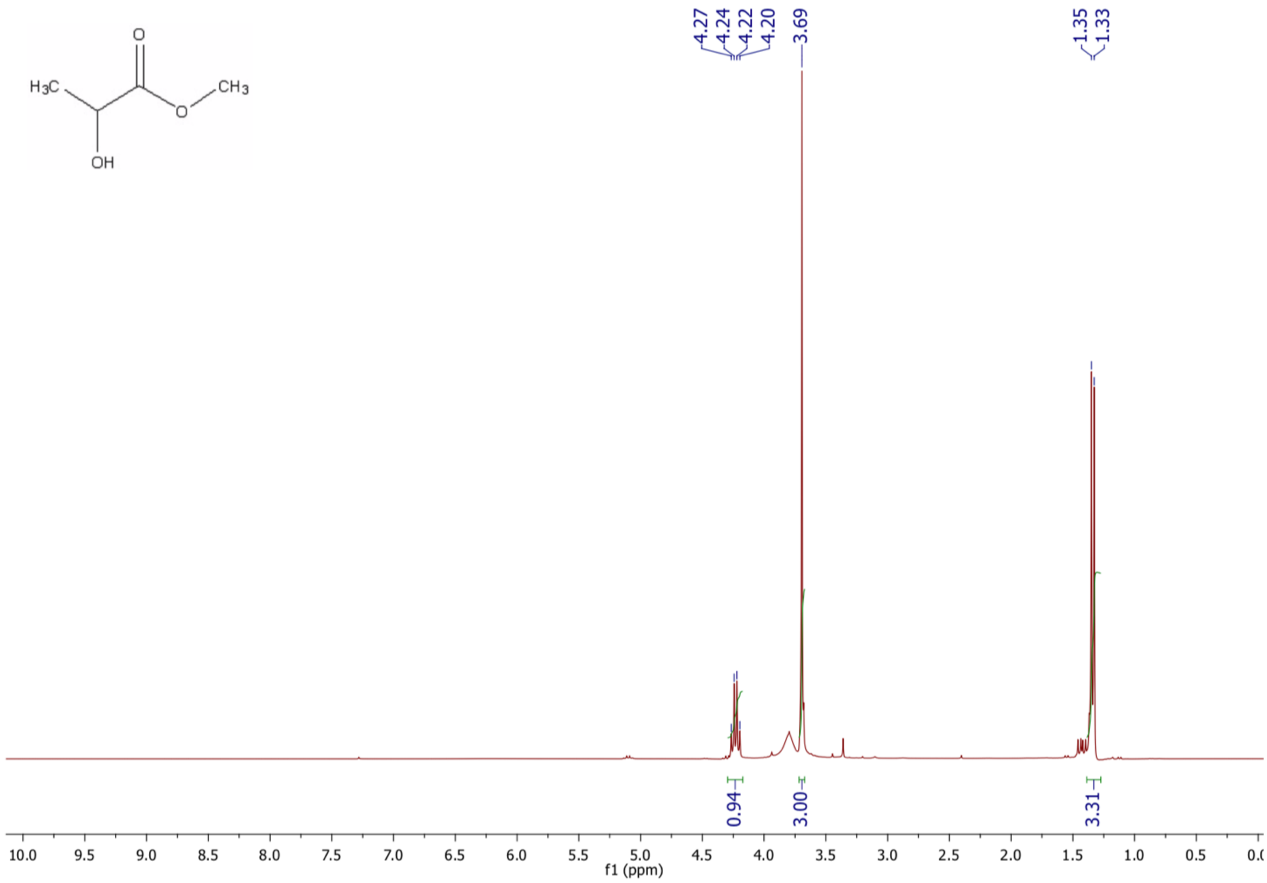
O
H3C
CH3
O
OH
4.27
4.24
4.22
4.20
3.69
1.35
1.33
0.94
3.00
3.31
10.0 9.5 9.0 8.5 8.0 7.5 7.0 6.5 6.0 5.5 5.0 4.5 4.0 3.5 3.0 2.5 2.0 1.5 1.0 0.5
f1 (ppm)

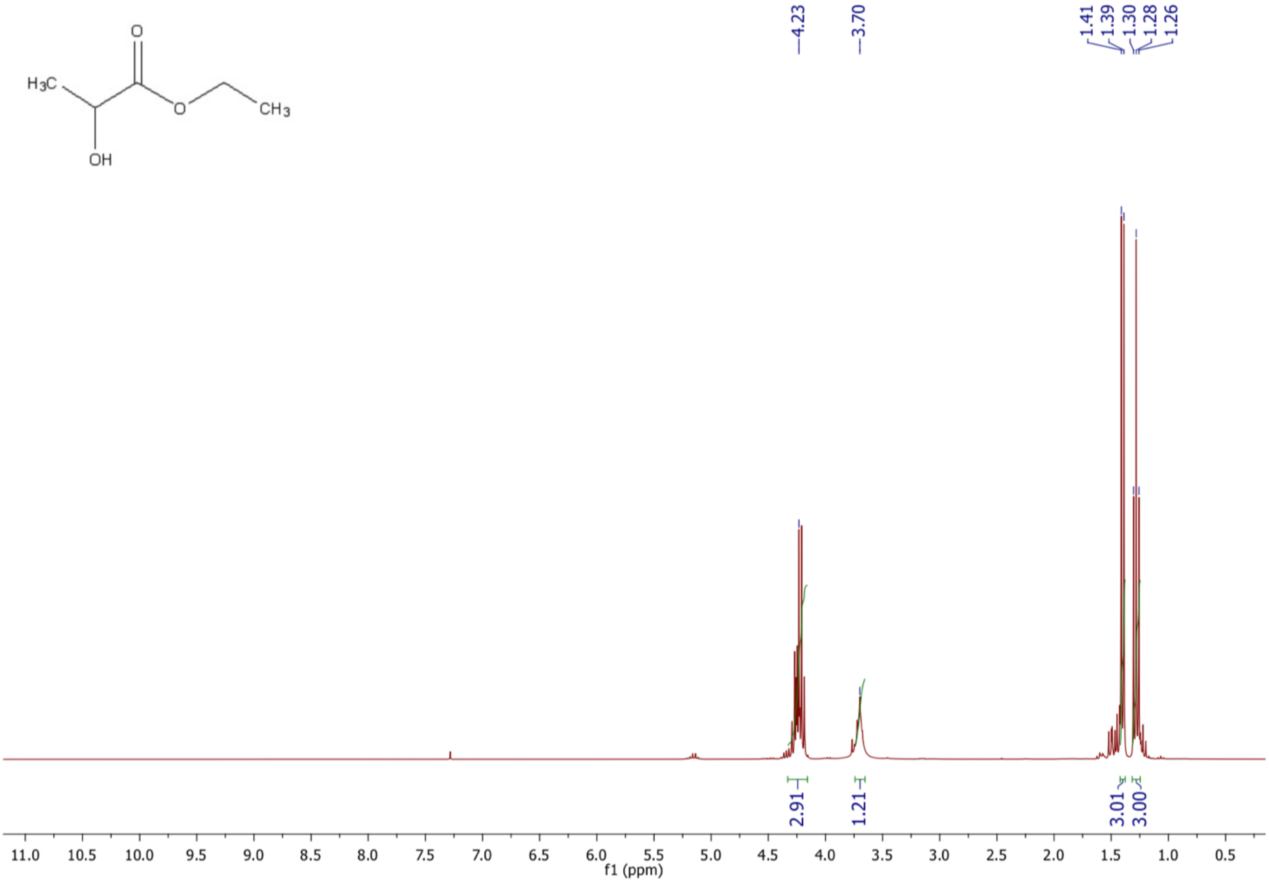
O
H3C
O
CH3
OH
4.23
3.70
1.41
1.39
1.30
1.28
1.26
2.91
1.21
3.01
3.00
11.0 10.5 10.0 9.5 9.0 8.5 8.0 7.5 7.0 6.5 6.0 5.5 5.0 4.5 4.0 3.5 3.0 2.5 2.0 1.5 1.0 0.5
f1 (ppm)

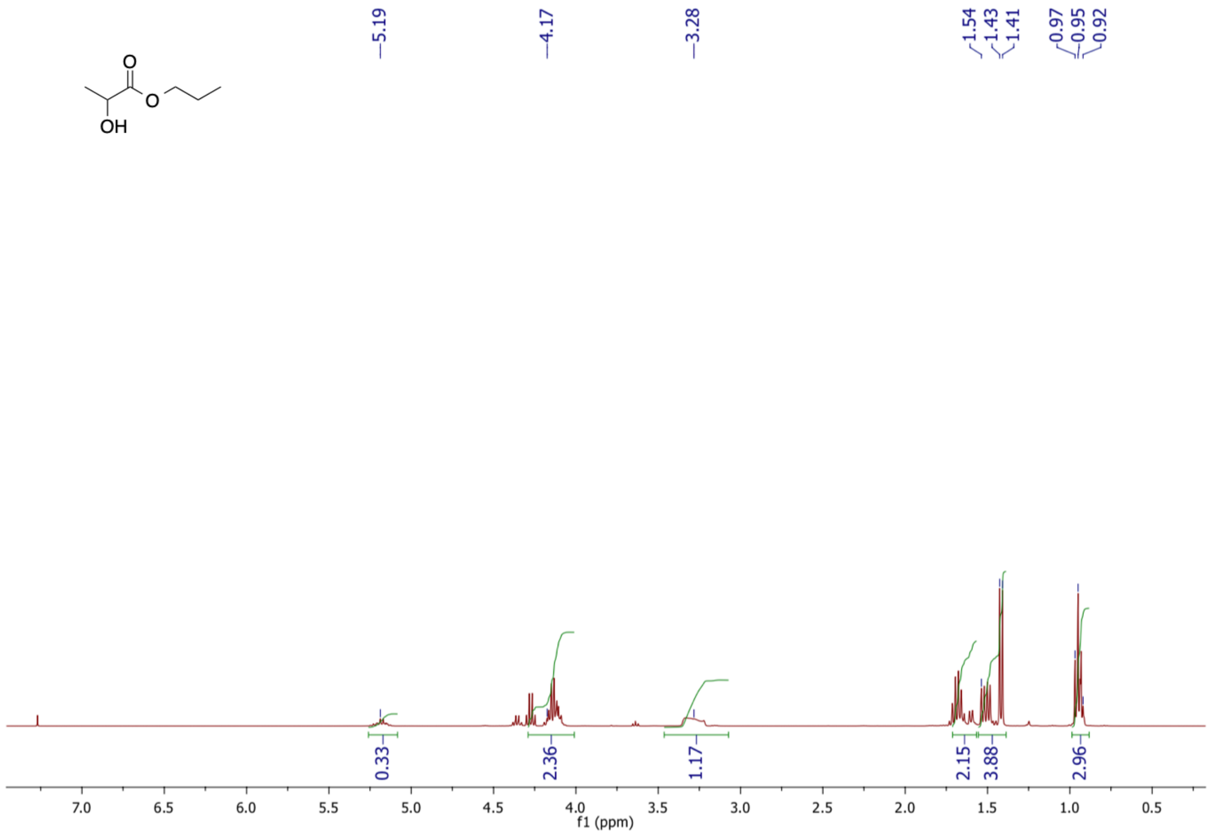
O
O
OH
5.19
4.17
3.28
1.54
1.43
1.41
0.97
0.95
0.92
0.33
2.36
1.17
2.15
3.88
2.96
7.0 6.5 6.0 5.5 5.0 4.5 4.0 3.5 3.0 2.5 2.0 1.5 1.0 0.5
f1 (ppm)

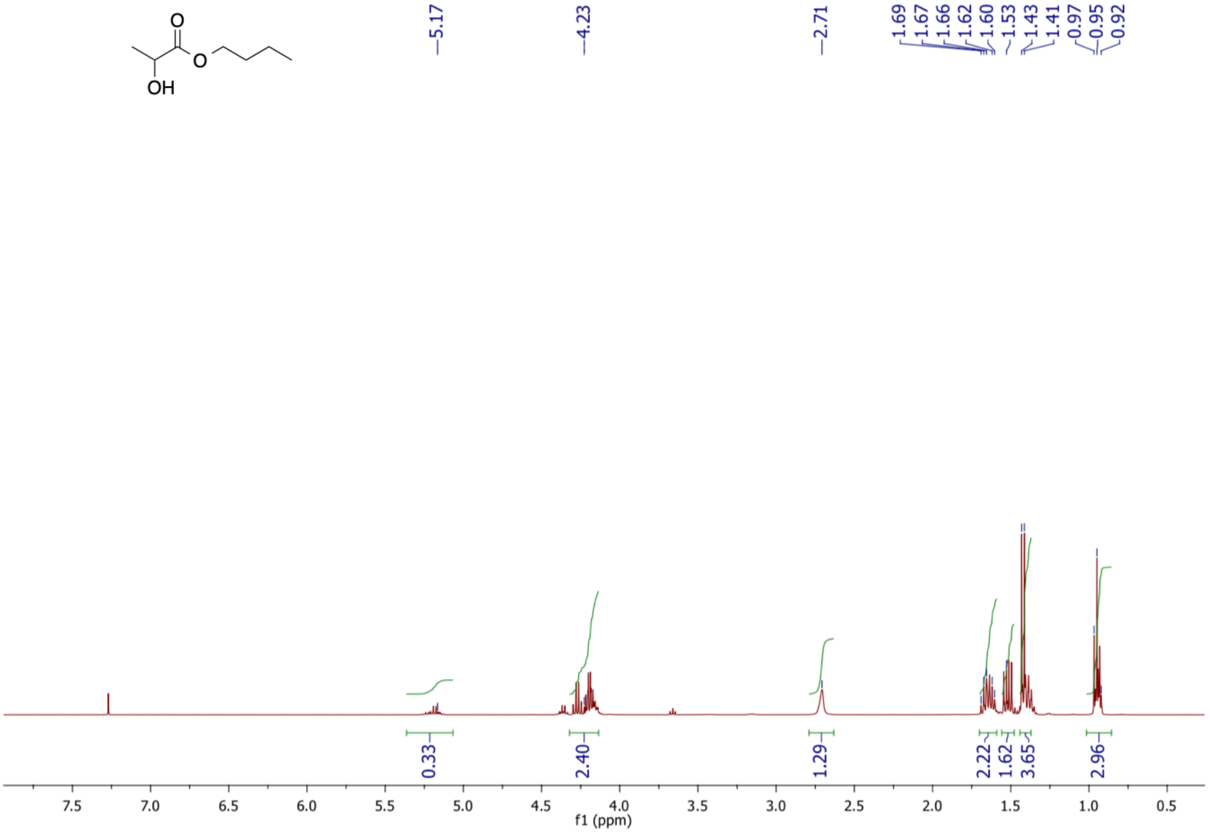
O
O
OH
5.17
4.23
2.71
1.69
1.67
1.66
1.62
1.60
1.53
1.43
1.41
0.97
0.95
0.92
0.33
2.40
1.29
2.22
1.62
3.65
2.96
7.5 7.0 6.5 6.0 5.5 5.0 4.5 4.0 3.5 3.0 2.5 2.0 1.5 1.0 0.5
f1 (ppm)

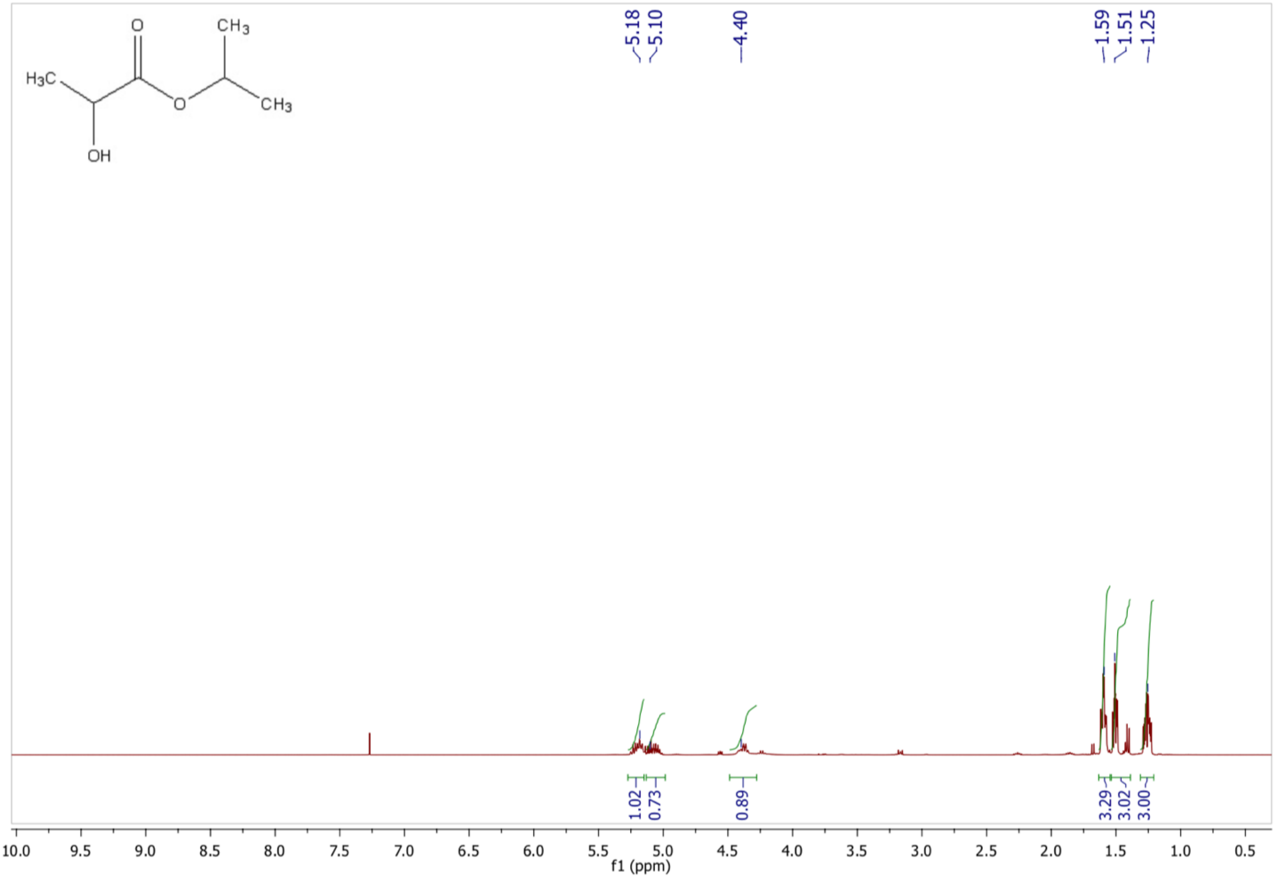

Figure S2. NMR spectra of lactate esters.

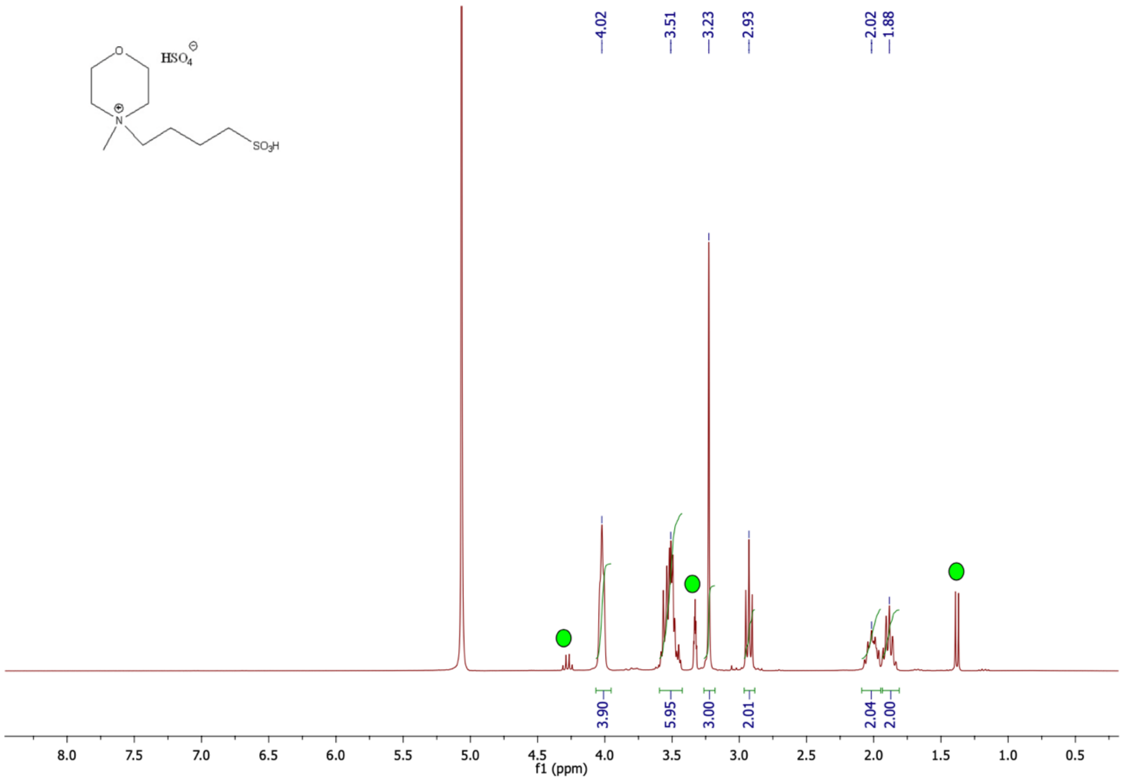

Figure S3. NMR spectrum of catalyst recovered after recycling. Peaks attributed impurities are marked .

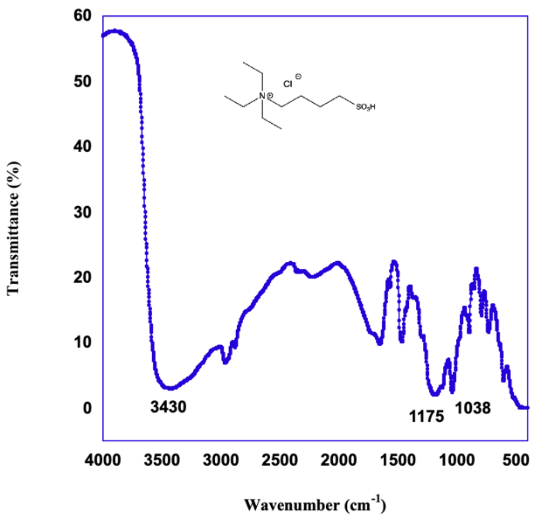
60
50
40
30
20
10
0
Transmittance (%)
Cl
SO3H
3430
1175
1038
4000
3500
3000
2500
2000
1500
1000
500
Wavenumber (cm-1)

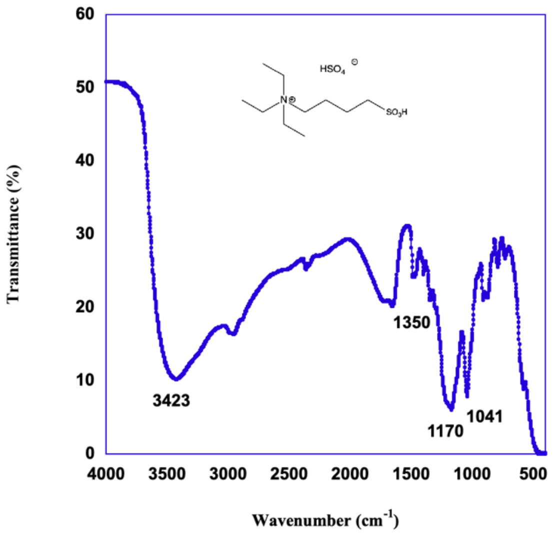
60
50
40
30
20
10
0
Transmittance (%)
HSO4
SO3H
3423
1350
1170
1041
4000
3500
3000
2500
2000
1500
1000
500
Wavenumber (cm-1)

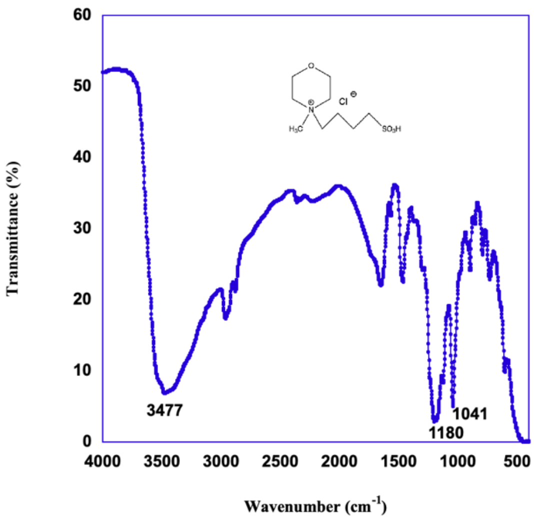
60
50
40
30
20
10
0
Transmittance (%)
Cl
H3C
SO3H
3477
1180
1041
4000
3500
3000
2500
2000
1500
1000
500
Wavenumber (cm-1)

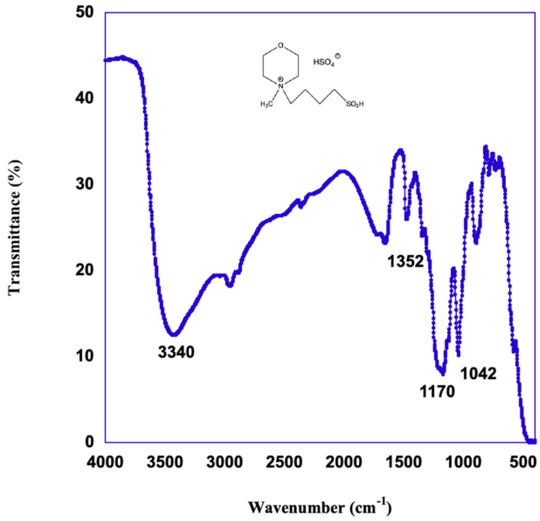
50
40
30
20
10
0
Transmittance (%)
HSO4
H3C
SO3H
3340
1352
1170
1042
4000
3500
3000
2500
2000
1500
1000
500
Wavenumber (cm-1)

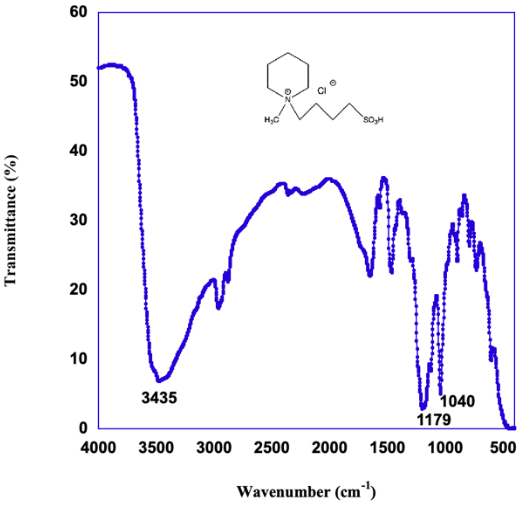
60
50
40
30
20
10
0
Transmittance (%)
Cl
H3C
SO3H
3435
1179
1040
4000
3500
3000
2500
2000
1500
1000
500
Wavenumber (cm-1)

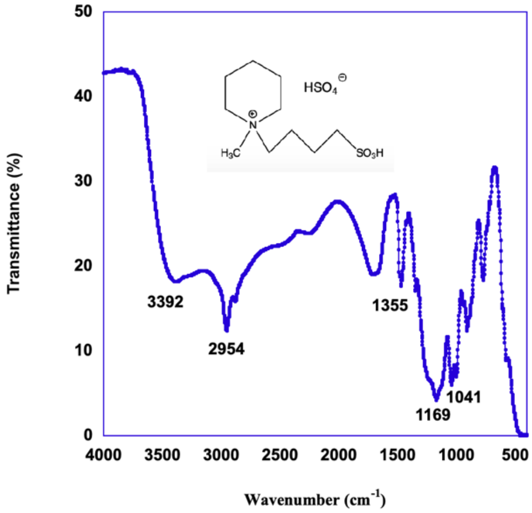
50
40
30
20
10
0
Transmittance (%)
HSO4
H3C
SO3H
3392
2954
1355
1169
1041
4000
3500
3000
2500
2000
1500
1000
500
Wavenumber (cm-1)

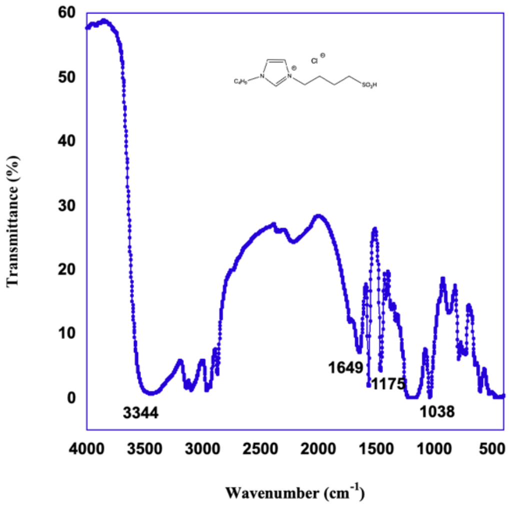

Figure S4. FTIR spectra of catalysts.

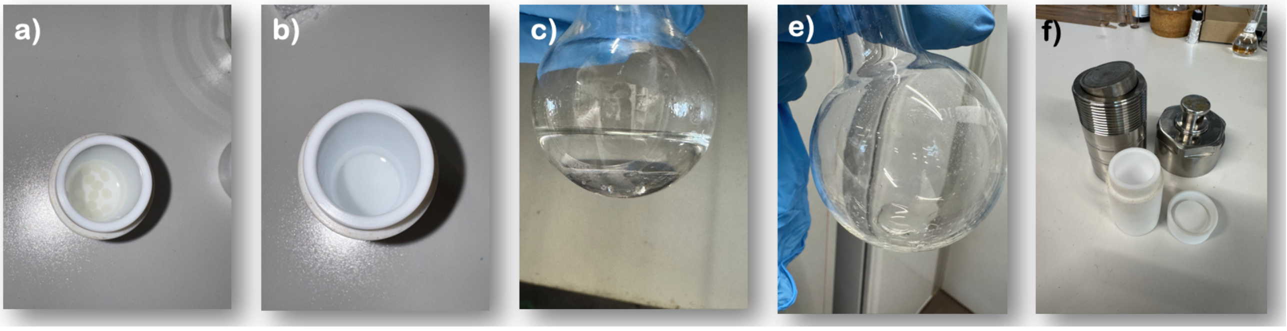

Figure S5. Representative pictures of a) Reaction mixture before alcoholysis reaction, b) reaction crude after alcoholysis reaction, c) biphasic extraction system after reaction, d) lactate ester isolated and e) hydrothermal reactor used.
